# Unusual mortality of Tufted puffins (*Fratercula cirrhata*) in the eastern Bering Sea

**DOI:** 10.1371/journal.pone.0216532

**Published:** 2019-05-29

**Authors:** Timothy Jones, Lauren M. Divine, Heather Renner, Susan Knowles, Kathi A. Lefebvre, Hillary K. Burgess, Charlie Wright, Julia K. Parrish

**Affiliations:** 1 School of Aquatic and Fishery Sciences, University of Washington, Seattle, Washington, United States of America; 2 Aleut Community of St. Paul Island Ecosystem Conservation Office, St. Paul, Pribilof Islands, Alaska, United States of America; 3 Alaska Maritime National Wildlife Refuge, U.S. Fish and Wildlife Service, Homer, Alaska, United States of America; 4 National Wildlife Health Center, U.S. Geological Survey, Madison, Wisconsin, United States of America; 5 Environmental and Fisheries Sciences Division, Northwest Fisheries Science Center, National Marine Fisheries Service, National Oceanic and Atmospheric Administration, Seattle, Washington, United States of America; Stockholms Universitet, SWEDEN

## Abstract

Mass mortality events are increasing in frequency and magnitude, potentially linked with ongoing climate change. In October 2016 through January 2017, St. Paul Island, Bering Sea, Alaska, experienced a mortality event of alcids (family: Alcidae), with over 350 carcasses recovered. Almost three-quarters of the carcasses were unscavenged, a rate much higher than in baseline surveys (17%), suggesting ongoing deposition and elevated mortality around St Paul over a 2–3 month period. Based on the observation that carcasses were not observed on the neighboring island of St. George, we bounded the at-sea distribution of moribund birds, and estimated all species mortality at 3,150 to 8,800 birds. The event was particularly anomalous given the late fall/winter timing when low numbers of beached birds are typical. In addition, the predominance of Tufted puffins (*Fratercula cirrhata*, 79% of carcass finds) and Crested auklets (*Aethia cristatella*, 11% of carcass finds) was unusual, as these species are nearly absent from long-term baseline surveys. Collected specimens were severely emaciated, suggesting starvation as the ultimate cause of mortality. The majority (95%, N = 245) of Tufted puffins were adults regrowing flight feathers, indicating a potential contribution of molt stress. Immediately prior to this event, shifts in zooplankton community composition and in forage fish distribution and energy density were documented in the eastern Bering Sea following a period of elevated sea surface temperatures, evidence cumulatively suggestive of a bottom-up shift in seabird prey availability. We posit that shifts in prey composition and/or distribution, combined with the onset of molt, resulted in this mortality event.

## Introduction

Climate change has been increasingly linked with shifts in marine ecosystem processes and structure [1–3]. In addition to long-term global warming [4] and large-scale modes of climatic variation (i.e. Pacific Decadal Oscillation [5]; North Atlantic Oscillation and El-Niño Southern Oscillation [6]), marine heatwaves (MHW)—prolonged periods of elevated sea surface temperatures (SST)–have emerged as a phenomena of ocean climate variability [7–8] that can significantly affect marine ecosystems [9, 10]. Although climate change is predicted to alter marine ecosystems globally, the effects of global warming are predicted to be the most extreme at higher latitudes [11].

The Bering Sea is a high latitude, semi-enclosed sea between the north Pacific and Arctic Oceans [12], notable for having an extensive continental shelf and seasonal ice-cover that varies in extent on interannual to multi-decadal time scales [2, 13]. Ecosystem structure, including the timing and composition of primary production and primary/secondary consumers, varies markedly among early and late ice-retreat years [14–19]. The eastern Bering Sea supports some of the most economically important fisheries in the world [1, 20], hosts large populations of marine mammals [21, 22], and is the breeding and/or summering ground for ~30–40 million marine birds [23–25]. Bering Sea food webs are particularly sensitive to bottom-up climate effects, as changes in atmospheric forcing impacts sea ice, as well as the extent of the ‘cold pool’, a lens of cold (< 2°C) near-bottom seawater that acts as a refuge for cold-water associated species, and also promotes primary production through summer/fall [18, 25, 26]. Over the last two decades, several multi-year stanzas of warm (2000–2005, 2014-present) and cold (2007–2013) conditions have been observed in the southern Bering Sea, which have been linked to variability in phytoplankton biomass (lower in warm years), copepod species composition (reduced abundance of large lipid-rich species in warm years) and forage fish energy density (lower in warm years) [25–27].

As abundant, visible, upper-trophic organisms, seabirds have been proposed as indicators of marine ecosystem shifts due to climate, with documented effects of climate variability on both reproduction [28–30] and adult survival [31–33]. Large-scale shifts in climate have been punctuated by large mortality events of marine birds [34–38]. These “massive mortality events” (MME)—defined as catastrophic, but often short-lived, periods of elevated mortality—can affect substantial proportions of a population, occasionally with long-term consequences to population size [39]. Seabird MMEs are perhaps one of the most frequently occurring and widely reported types of MME in the literature [40], potentially due to their perceived and absolute (mortality often exceeding 10,000s-100,000s birds; [35, 38, 40, 41]) magnitude.

**Fig 1:**
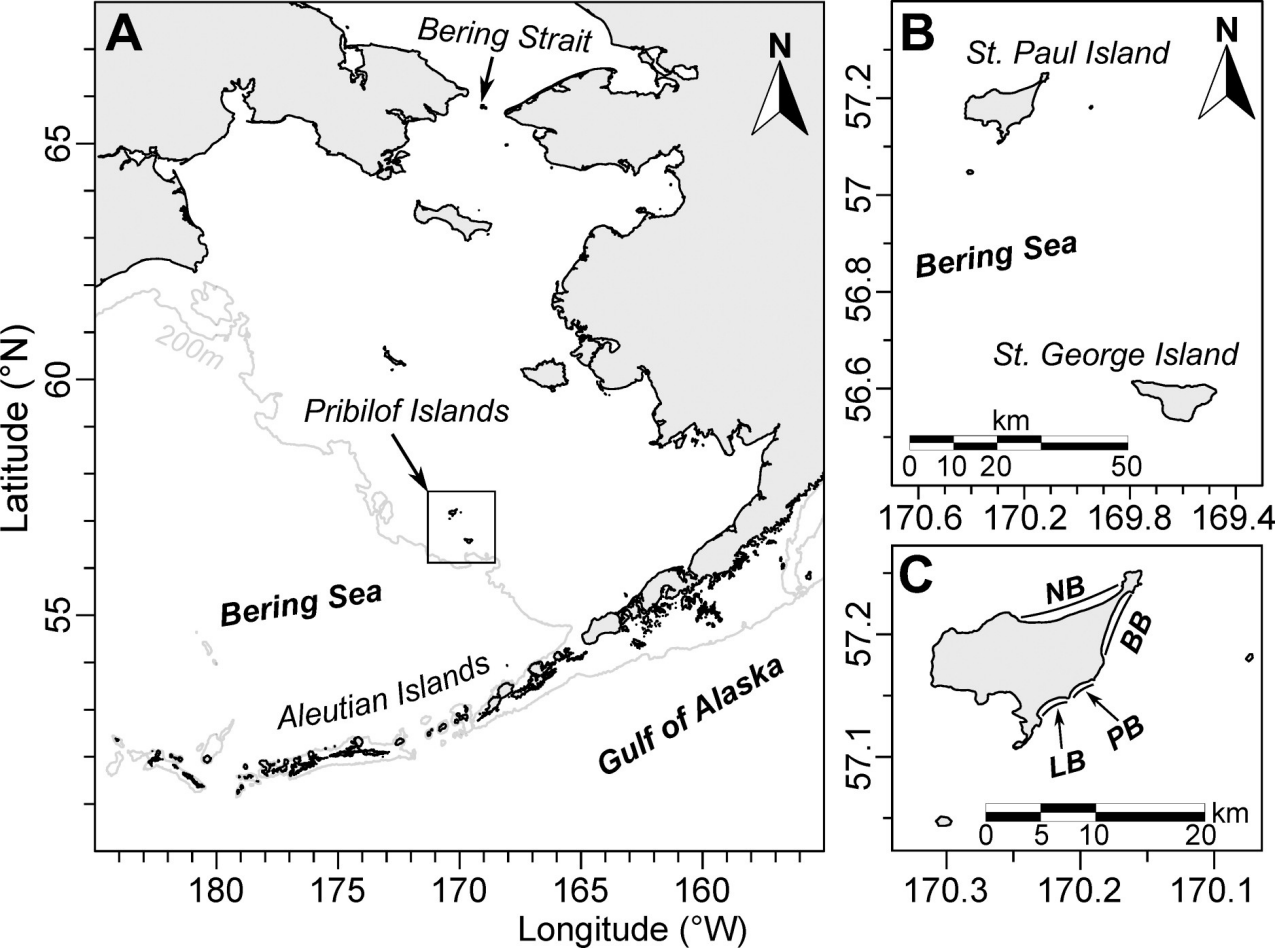
Location of St Paul Island within the Pribilof Island group in the Bering Sea. (A) The Bering Sea centered on the Pribilof Islands, Alaska, with the 200m (shelf break) isobath shown. (B) St. Paul and St. George Islands in the Pribilof Islands. (C) Surveyed beaches on St Paul Island: NB–North Beach, BB–Benson Beach, LB–Lukanin Beach, PB–Polovina Beach.

In this paper, we document a MME of marine birds, primarily Tufted puffins (*Fratercula cirrhata*), on St. Paul Island, Pribilof Islands, Alaska, in the eastern Bering Sea (**Fig 1**) during the late fall/winter of 2016/2017. The Pribilof Islands, located near the edge of the Bering Sea continental shelf, support one of the largest concentrations of breeding seabirds (>2 million) in the North Pacific [42, 43]. The islands have also been hunting and harvesting grounds to Unangan (or Aleut) for millennia, with permanent settlements on both islands established in the late 1700s. Several species of seabirds are important cultural and subsistence resources [44], and as such seabird mortality events are both an ecological and societal concern for island residents. Using a combination of long-term standardized beached bird surveys and intensive surveys during the event, we characterize this MME in terms of timing, abundance, species composition and carcass condition as compared to baseline measures. We use wind forcing and carcass count phenology to model daily deposition and provide estimates of total mortality. Our results add to the growing body of literature documenting marine bird MMEs in the northeast Pacific associated with recent and persistent warming [10, 38, 45].

## Methods

### Data collection

Beached bird data on St. Paul Island (SPI) including the date, location, taxonomic identification, condition and effort-controlled count were collected by the Aleut Community of St. Paul Island Ecosystem Conservation Office (ACSPI-ECO) in collaboration with the Coastal Observation and Seabird Survey Team (COASST). COASST is a citizen science program in which trained participants conduct monthly standardized surveys, recording all new and previously observed carcasses within prescribed beaches. Field identifications are made from recorded morphological evidence (foot type; standardized body measurements) and consultation with a bird identification guide [46]. Carcasses are individually marked, photographed and subsequently verified by experts using morphological and photographic evidence. Bi-weekly to monthly surveys have been carried out on four 1 km beaches on SPI (**Fig 1**) since 2006. Additional COASST surveys from nearby St. George Island (~80 km distant; SGI; 2 beaches), as well as throughout the Aleutian Islands (14 beaches on 5 islands), provide a baseline (inclusive of surveys conducted 2006–2015) of effort-standardized carcass abundance and taxonomic composition.

**Fig 2:**
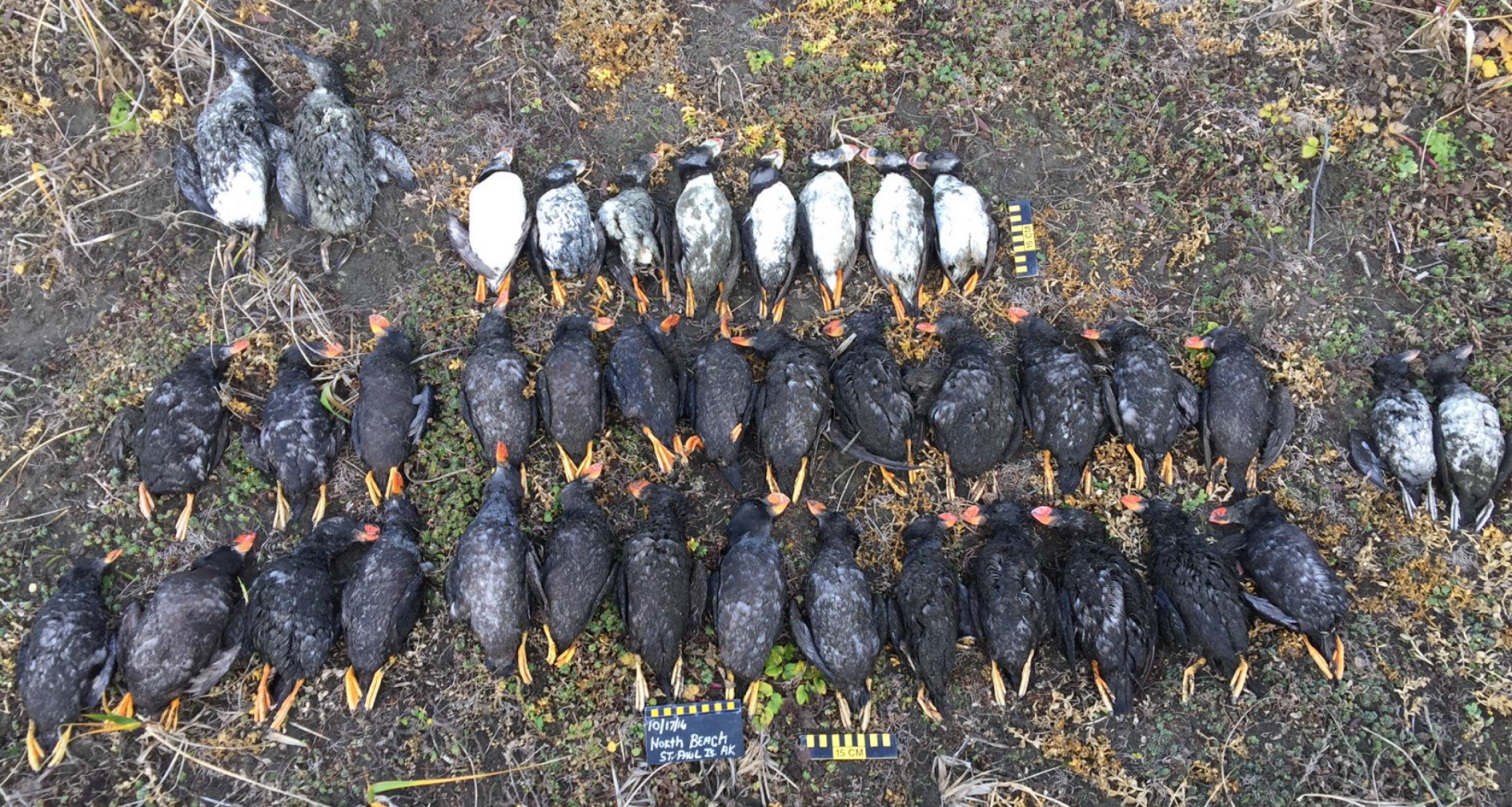
Photo of carcasses found on North Beach, St. Paul Island, Alaska, on 17 October 2016. Birds pictured are 2 murres (*Uria* spp.—top row left corner), 8 Horned puffins (*Fratercula corniculata*—top row center), 2 juvenile Tufted puffins (middle row right side) and 27 adult Tufted puffins (middle and bottom rows). Scale bar in photo is 15 cm total length.

During the 2016 MME, extremely high numbers of carcasses and difficult weather conditions necessitated the development of a streamlined protocol (COASST *Die-Off Alert*). Created and tested by ACSPI-ECO and COASST, the *Die-Off Alert* protocol requires collection and removal of all carcasses on a set length of beach to a safer location off the beach where they are sorted by species, age class, and carcass condition (i.e. intactness), and photographed in groups (**Fig 2**). Although primary evidence (e.g. measurements, body condition) is not recorded, the *Die-Off Alert* protocol facilitates collection over a larger beach area, and gross anatomical features (e.g. intactness, molt) are visible from photographs, enabling post-collection verification.

### Necropsies

Eight intact carcasses (6 Tufted puffins—5 adults, 1 juvenile, and 2 adult Horned puffins–*Fratercula corniculata*) collected on SPI in October 2016 were sent to the National Wildlife Health Center (USGS). Tissues collected for histopathology (5 birds) were fixed in 10% neutral buffered formalin, processed routinely, embedded in paraffin and sectioned at approximately 5 μm. Routine bacterial cultures of the liver were conducted on five birds. Tracheal and cloacal swabs from all birds were tested for avian influenza [47], and feather pulp was tested for avian paramyxovirus-1 (2 birds) [48] and for West Nile virus (1 bird) [49]. Cloacal contents (4 birds) and stomach content samples (2 birds) were sent to the Wildlife Algal-toxins Research and Response Network (WARRN-West) to analyze for harmful algal toxins domoic acid and saxitoxin using an ELISA (Abraxis, Inc.). Remaining birds could not be tested for algal toxins due to insufficient stomach contents for diagnostic analyses, or decomposition state [50].

### Quantifying event versus baseline

We conducted two quantitative comparisons of event versus baseline data: lineal carcass encounter rate (ER—carcasses per km of beach surveyed) and taxonomic composition. Monthly baseline ER was calculated as the average for all month-years of data available for the Pribilof Islands (SPI and SGI, 6 beaches). Ranges in baseline estimates were calculated as the bootstrapped 95% confidence interval (95% CI) of the mean for each calendar month. To create a broader geographic comparison, we extended taxonomic comparisons to data collected on the Aleutian Islands (14 beaches on 5 islands).

### Particle trajectory modeling

To determine the likely origin of carcasses at sea (e.g. catchment area) and to estimate proportional beaching rates, we ran a series of wind-forced particle simulation experiments. Daily releases of 10,000 surface-trapped particles (i.e. replicating a floating bird carcass) were simulated according to wind conditions observed from 1 October 2016 to 24 November 2016, capturing the period where the majority of carcasses were deposited on SPI. Because we had no *a priori* knowledge of the at-sea distribution of birds prior to the mortality event other than the occurrence of beached birds on SPI, but not on SGI, we randomly generated starting particle locations at a uniform density around SPI, with initial distances, *d*_*0*_, up to 100 km. A maximum distance of 100km from SPI was chosen as it results in simulated deposition on SGI, allowing us to identify maximal and closer distributions where deposition on SGI would have been minimal.

Previous studies have found strong correlations between carcass deposition and wind-speed and direction [51–54]. We obtained wind velocity fields from the North American Regional Reanalysis (NARR) dataset [55], which consists of 3-hourly averaged grids (32 km resolution). Particle movement from one time-step to another (i.e. 3 hours) was modeled using 4^th^ order Runge-Kutta numerical integration, assuming particle windage equal to 2.5% of the location (linearly interpolated from the nearest 4 NARR grid points), and time-specific wind velocity [51, 52, 56, 57]. Although local surface currents likely contribute to carcass dispersal, we are unaware of surface current information resolved at a suitable temporal (3-hourly) scale to capture nearshore current dynamics. Particle trajectories were simulated from release until intersection occurred with the coastline of SPI or SGI, or 14 days [54], whichever came first.

To account for sinking, each particle intersecting either island was assigned a probability of reaching shore, modeled as a logistic function of float duration [54]:

$$p(f)=\eta_{1}-\left(\frac{\eta_{1}}{1+e^{-\eta_{2}(f-\eta_{3})}}\right)$$ (Eq 1)

where *p*(*f*) is the proportion of carcasses remaining afloat as a function of float duration, *f*, in hours. Parameters *η*_1−3_ control the shape (i.e. rate and mid-point) of the float function, and determine the rate at which simulated carcass sinking occurs (modeled after [54]). Values for *η*_1−3_ were specified to match observations of carcass float duration from Alaska, where cooler temperatures may delay decomposition, allowing carcasses to remain afloat longer [54, 58]. Ford et al. [58] found median float durations of 7 and 9 days in Prince William Sound, Alaska, with nearly all carcasses sunk by 14 days. We specified two alternate sink functions, with median float durations of 7 and 9 days (**S1 Fig**), and tested among them to determine how alternate representations of float duration affected our mortality results. For particles remaining at sea for the entire 14 days, *p*(*f*) was set to zero.

### Catchment analyses

To identify catchment area, or the area of ocean within which carcasses could have originated based on observed deposition, we focused on three temporal windows of carcass deposition: (1) 17 to 21 October 2016, (2) 27 October to 1 November 2016, and (3) 15 to 23 November 2016, as these three periods had consistent survey effort, differential patterns of carcass deposition, and differential wind direction (north versus south; **S2 Fig**). For each window, we took release sets ranging from three days prior to the first MME date (70% of simulated deposition occurs within 3 days–**S3 Fig**) to the end of the MME window (inclusive) and calculated a grid of proportional deposition (5×5 km grid cells arrayed from SPI to 100 km offshore) by summing *p*(*f*) for particles originating in each grid cell that ‘beached’ on SPI and SGI, respectively.

In the absence of at-sea distribution data for birds prior to mortality, we assumed that birds were uniformly distributed around SPI out to a maximum distance, *d*_*max*_, which we varied (i.e. by subsetting all particle releases) to investigate alternate spatial distributions. For a given *d*_*max*_ we estimated proportional deposition on SPI and on SGI for all particles with initial distance, *d*_*0*_ ≤ *d*_*max*_, for each of the three observed deposition time windows. To explore the relationship between offshore distribution and island-specific proportional deposition, we varied *d*_*max*_ from 2 km to 100 km. Calculating the ratio of proportional deposition on SGI relative to SPI as a function of *d*_*max*_ allowed us to bound at-sea distributions by identifying the value of *d*_*max*_ at which deposition on SGI would have been expected given deposition on SPI. We also calculated proportional deposition on day *i* from release *j*, *P*_*j*_*(i)*, by summing *p*(*f*) for particles deposited on day *i*, from release *j*. Alternate time-series of expected deposition on SPI and SGI per day (i.e. ∑_*j*<*i*_*P*_*j*_(*i*)) were then calculated for alternate values of *d*_*max*_. We explored the likelihood of alternate at-sea distributions (proxied by *d*_*max*_) by calculating the ratio of simulated deposition on SGI relative to SPI for different values of *d*_*max*_. This allowed us to identify those distributions that would have resulted in minimal expected deposition on SGI, versus those where expected deposition rates would have been comparable among islands. For daily proportional deposition rates we restricted this analysis to time windows when deposition occurred on SPI as these were time intervals when carcasses were known to be afloat, and therefore could have been deposited on SGI.

### Total mortality estimation

Total mortality estimates are dependent on observed carcass abundance, survey effort, and estimates of carcass detection, persistence and proportional beaching rates. We assumed that counts, *C*_*b*,*d*_, made on beach, *b*, on day, *d*, were equal to the sum of deposition following the previous survey on that beach, minus the proportion that are washed away or scavenged, plus the number of carcasses missed in the previous survey on day *d’* that remained on the beach:

$$C_{b,d}=(1-\varphi )\rho (d-d^{\prime })C_{b,d^{\prime }}+\varphi L_{b}\sum_{i=d\prime +1}^{d}\rho (d-i)D_{i}$$ (Eq 2)

where *φ* is detection rate, *L*_*b*_ is length of beach suryeved, *ρ*(*d*) is the proportion of carcasses remaining as a function of time since deposition (i.e. carcass persistence), and *D*_*i*_ is the daily rate of carcass deposition per km [41]. Daily deposition rate, *D*_*i*_, was modeled using expected proportional deposition rates *P*_*j*_*(i)* from particle simulations. Because of the predominantly NW/S wind directions (**S4 Fig**), we split SPI into northern and southern halves and calculated *P*_*j*_*(i)* for each half separately. Daily deposition rate, *D*_*i*_, is the sum of proportional deposition, multiplied by an effective mortality rate *M*_*j*_:

$$D_{i}=\frac{1}{L}\sum_{j=i-14}^{j=i}M_{j}P_{j}(i)$$ (Eq 3)

where effective mortality rate is the number of carcasses required to sustain deposition that when summed over time meet the observed carcass counts. The fraction 1/*L* converts from island-wide deposition to deposition per km, where *L* was held constant at 21.8 km, equal to the maximum linear dimension (NE to SW) of SPI, effectively modeling the area presented to the wind by the northern/southern halves of the island.

Using our observed counts, *C*_*b*,*d*_, (Eq 1) we calculated a range of estimates for *M*_*j*_, making the assumption that effective daily mortality rates were constant across releases that contributed to each count (i.e. $M_{j}=\tilde{M}$). Combining Eqs 2 and 3 results in an effective daily mortality rate, $\tilde{M}$, from survey counts as:

$$\tilde{M_{b,d}}=\frac{L}{\varphi L_{b}}{\left(\sum_{i=d^{\prime }+1}^{i=d}\rho (d-i)\sum_{j=i-14}^{j=i}P_{j}(i)\right)}^{-1}[C_{b,d}-((1-\varphi )\rho (d-d^{\prime })C_{b,d\prime })]$$ (Eq 4)

Because we had no event-specific data on detection (*φ*) or persistence (*ρ*) rate we selected relevant published values, and examined the sensitivity of mortality estimates to variations in these assumptions. A study on SPI in February 1996 [59] found that daily carcass persistence was lower in the first 24 hours, and higher on subsequent days, with other studies coming to similar conclusions [60, 61]. Therefore, we modeled persistence with respect to carcass residence time on the beach, *τ*, as a two-stage process:

$$\rho (\tau )=\left\{ \begin{array}{l} \rho_{0}{\rho_{1}}^{\tau -1}\;if\;\tau >0\\ 1\;if\;\tau =0 \end{array}\right.$$ (Eq 5)

where *ρ*_0_ is the proportion that remain one day after deposition and *ρ*_1_ is the daily persistence rate on subsequent days. Using first day (0.72–0.79) and subsequent (0.85–0.94) average daily persistence rates from [59], recorded on SPI during winter, we specified that *ρ*_0_ was normally distributed with 0.72 and 0.79 as ± 1 sd from the mean (*ρ*_0_~*N*(0.755,0.035)), and *ρ*_1_ was normally distributed with 0.85 and 0.94 as ± 1 sd from the mean (*ρ*_1_~*N*(0.895,0.045)) to encapsulate the uncertainty regarding these values (see **S5 Fig**).

Previous studies have reported a range of values for carcass detection rates (0.41–0.7 [61]; 0.79–0.88 [62]; 0.42–0.6 [63]). Given deteriorating environmental conditions prevalent during the mortality event we assumed that detection rate was most similar to the two studies that reported lower detection rates (i.e. [61] and [63]). Averaged detection rate from those studies was 0.53, but given the uncertainty around this parameter, we specify that detection rate was normally distributed with 0.4 and 0.66 (i.e. approximately the average upper and lower values reported from those studies) as ± 1 sd from the mean (*φ*~N(0.53,0.13)). Although we were unable to estimate carcass persistence and detection rates from baseline surveys, refind rates from baseline surveys on SPI (12%, N = 58 birds, survey interval = 11–15 days), compared favourably with these assumptions (median persistence at day 13 = 0.23 × detection rate of 0.53 = refind rate of 12%).

We constructed an estimate of mortality by randomly drawing values for persistence (*ρ*_0_,*ρ*_1_) and detection (*φ*) in order to calculate $\tilde{M_{b,d}}$ according to Eq 4. We multiplied the resultant average daily mortality rate by the duration of the event, which we conservatively define as 12-October to 23-November (N_day_ = 43). We define the start of the mortality event as the 12-October because although carcasses could have been afloat for days prior to deposition (i.e. the mortality event started earlier), we have no knowledge of at-sea distribution or float duration to inform this, and so we conservatively set the start of the mortality event one day prior to the first report of beachcast birds. After 23 November 2016, surveying became sporadic due to adverse weather conditions such that mortality could not be reliably estimated. We then repeated this procedure 5,000 times, with each permutation based on random draws of persistence and detection rates, in order to create a distribution of mortality estimates according to model parameter uncertainty. The entire procedure was repeated for each scenario of maximum distance offshore (defined from catchment analyses) and float duration that affected the proportion of carcasses deposited (*P*_*j*_(*i*) in Eq 4), in order to examine how our mortality estimates varied with the assumption of offshore distribution.

All analyses were carried out in R version 3.4.3 [64].

## Results

### MME description

Carcasses were first encountered by residents on 13 October 2016. Standardized surveys began on 17 October and continued into February 2017. We placed surveys into three time periods based on survey frequency and weather: 17 October to 1 November 2016 (N_surv_ = 13); 15 to 23 November 2016 (N_surv_ = 6); and 7 December 2016 to 3 February 2017 (N_surv_ = 6).

**Fig 3:**
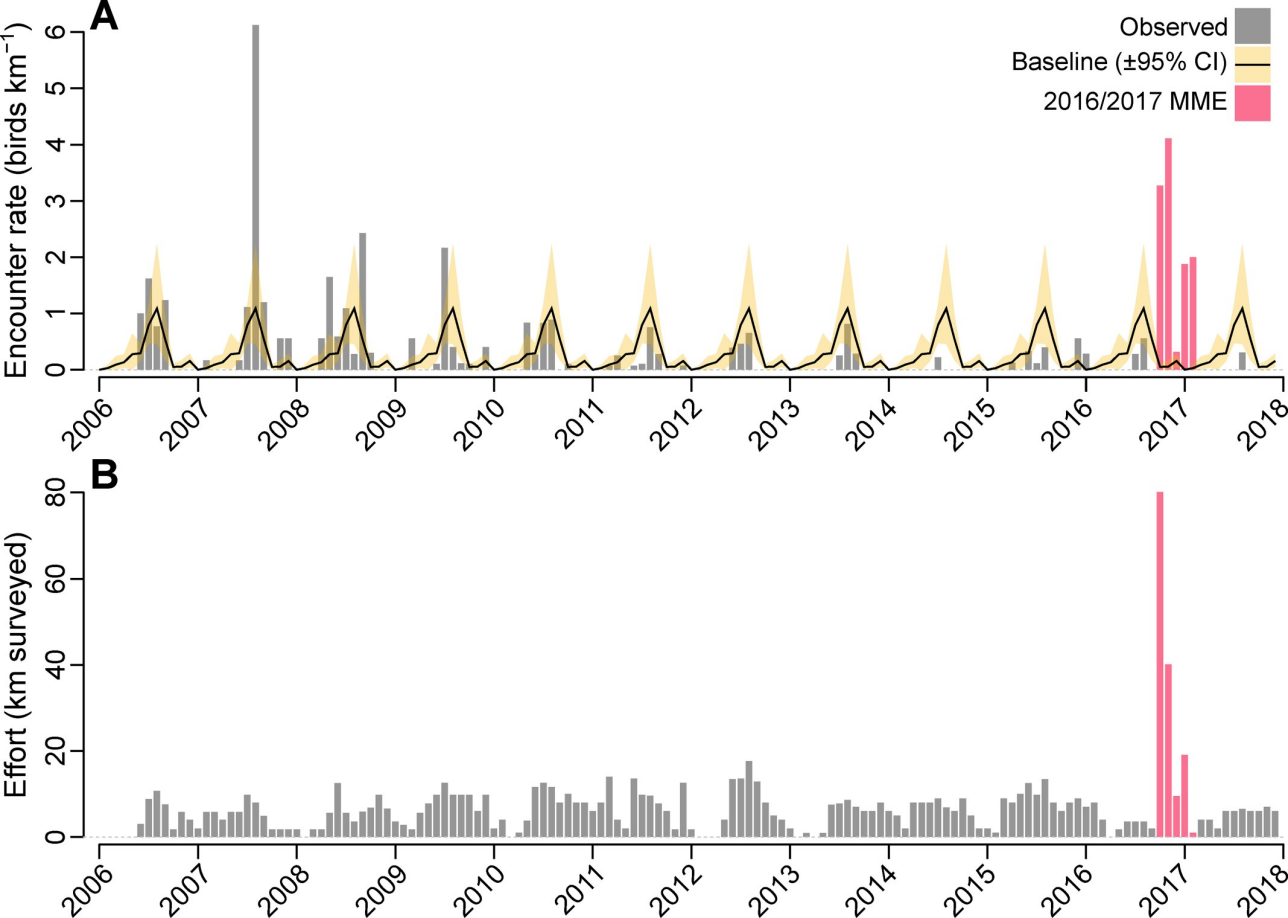
Time-series of effort-standardized beached bird abundance and survey effort on the Pribilof Islands, Alaska. (A) Month-averaged relative carcass abundance (carcasses km^-1^) for the Pribilof Islands, plotted as a function of time, with baseline average (monthly from 2006 to 2015 inclusive, ±95% CI) overlaid. (B) Cumulative survey effort per month for the Pribilof Islands, plotted as a function of time. The months of the 2016/2017 event are highlighted in red.

During the first period, 247 carcasses were found, consisting of Tufted puffins (88%), primarily adults (93% of Tufted puffins with determinable age, N = 211), Horned puffins (6%) and Common or Thick-billed murres (*Uria* spp.) (6%; **Table 1**). All-species encounter rates were 65 times higher than the Pribilof Islands baseline (ER = 3.27 versus 0.05 carcasses km^-1^, 95% CI 0.03–0.12; **Fig 3**) during this time period. During the second period (mid-November) an additional 78 carcasses were recorded, again mostly Tufted puffins (83%), with Crested auklets (*Aethia cristatella*) making up the remainder (15%), and an average encounter rate 74 times higher than baseline (ER = 4.11 versus 0.05 carcasses km^-1^ 95% CI 0.02–0.18; **Fig 3**). In the third and final period, surveys had become sporadic due to weather. By January (N_surv_ = 3), Crested auklets were the only species found (**Table 1**). Throughout the event, a large proportion (72%) of recovered carcasses were fresh and unscathed (e.g., intact carcass with clear fully rounded eyes). Baseline SPI surveys, by contrast, had considerably lower rates of intactness (17%, N = 176; see **S**1 **Text**). Carcass abundance and relative intactness suggest that recent mortalities were deposited on a daily basis from mid-October to at least mid-November, indicating that mortality was ongoing throughout that period (**Table 1**).

**Table 1:** Observed counts made on St. Paul Island, Alaska, from October 2016 through to January 2017.

Date	Beach^a^	Length (km)	TUPU (A)^b^	TUPU (J)^b^	TUPU (A/J)^b^	HOPU^b^	CRAU^b^	Murre	Total	N:S^c^ (%)	% intact
17-Oct	NB	10	27	2	0	8	0	2	**39**	100:0	92.3
18-Oct	NB, BB	16.5	6	1	1	0	0	0	**8**	63:37	100
19-Oct	NB	10	16	3	0	2	0	1	**22**	100:0	63.6
20-Oct	NB	10	16	4	1	4	0	1	**26**	100:0	46.2
21-Oct	NB	10	12	4	0	2	0	0	**18**	100:0	61.1
25-Oct	LB, NB	13.1	4	0	0	0	0	0	**4**	75:25	25.0
27-Oct	BB, LB, PB	11.5	72	0	4	0	0	7	**83**	0:100	85.5
1-Nov	LB, PB	5	43	1	0	0	0	3	**47**	0:100	68.1
15-Nov	BB, NB	16.5	37	0	0	0	8	0	**45**	80:20	60.0
18-Nov	LB, PB	5	10	0	0	0	1	1	**12**	0:100	0
23-Nov	NB, BB	16.5	17	0	1	0	3	0	**21**	71:29	57.1
7-Dec	NB, BB	16.5	2	0	1	0	3	0	**6**	100:0	33.3
3-Jan	NB, BB	16.5	0	0	0	0	13	0	**13**	38:62	100
11-Jan	LB	3.1	0	0	0	0	13	0	**13**	0:100	84.6
3-Feb	LB	1.0	0	0	0	0	0	2	**2**	0:100	0
**Total**		**161.2**	**262**	**15**	**8**	**16**	**41**	**17**	**359**	**49:51**	**69.6**

^a^ Beach names: NB—North Beach, BB—Benson Beach, LB—Lukanin Beach, PB—Polovina Beach

^b^ Species: TUPU—Tufted puffin, HOPU—Horned puffin, CRAU—Crested auklet, A—adult, J—juvenile.

^c^ N:S north:south ratio of carcass deposition observed on St Paul Island for that survey date.

**Fig 4:**
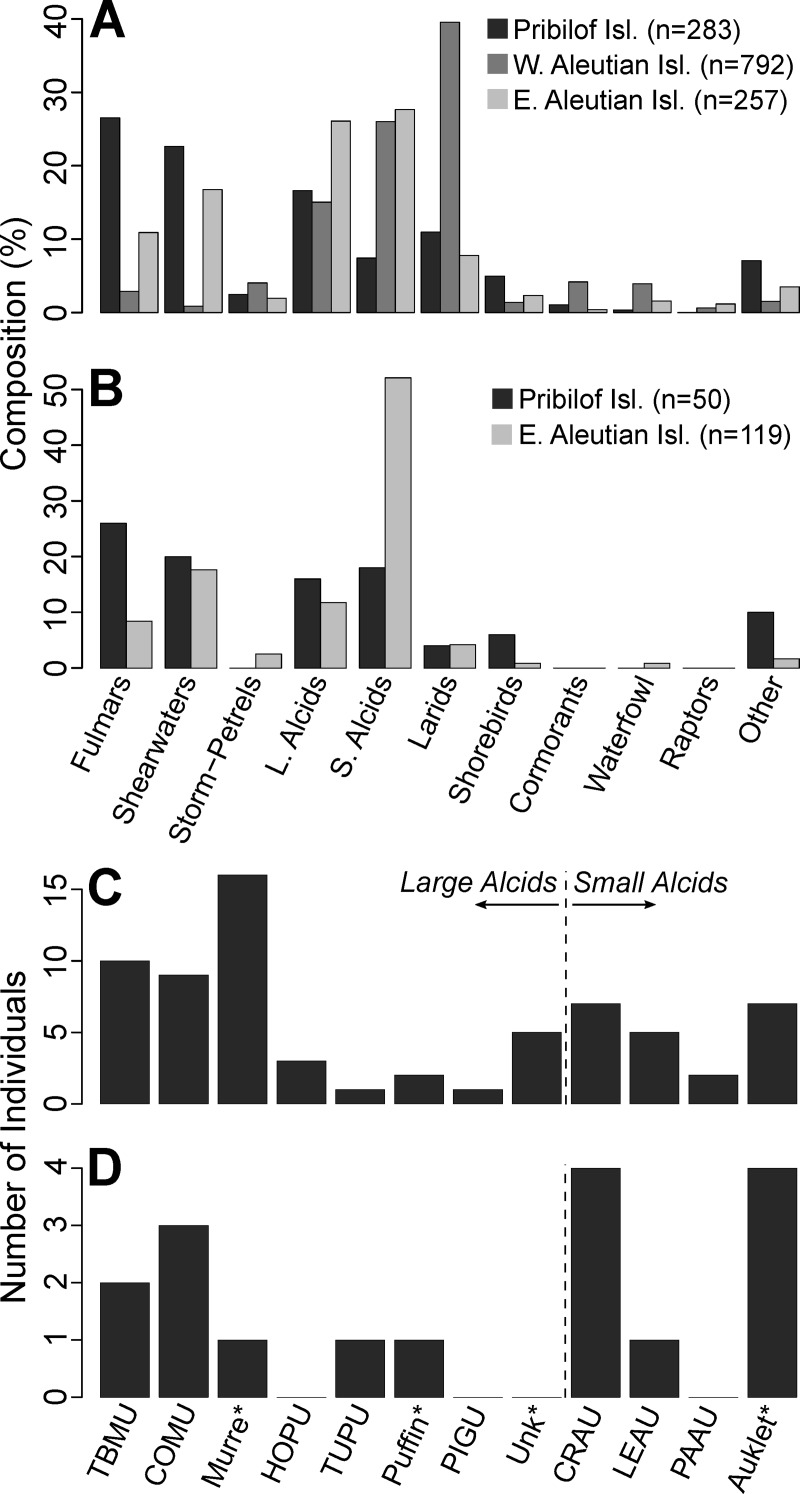
Baseline taxonomic composition of beached bird carcasses found on the Pribilof Islands and Aleutian Islands. Species composition is shown year round (A) and for the fall/winter period (B—September to December) for all taxonomic groups recorded on the Pribilof Islands, Alaska and the eastern and western Aleutian Islands, Alaska. Fall/winter data were unavailable from the Western Aleutian Islands. Alcid species composition is shown for the Pribilof Islands year round (C) and for the fall/winter period (D). Asterisks (*): carcasses identified to group but not species. TBMU: Thick-billed murre (*Uria lomvia*), COMU: Common murre (*Uria aalge*), HOPU: Horned puffin, TUPU: Tufted puffin, PIGU: Pigeon guillemot (*Cepphus columba*), CRAU: Crested auklet, LEAU: Least auklet, PAAU: Parakeet auklet (*Aethia psittacula*).

Regular monitoring on the Pribilof Islands recorded elevated beaching rates from July to September of 2007 (**Fig 3**), primarily Short-tailed shearwaters (64%, N = 66) and Northern fulmars (15%) (**S3 Table**), consistent with the usual species composition and phenology of beached bird abundance on the Pribilof Islands (i.e. **Fig 3** and **Fig 4**). Prior to the 2016/17 event, COASST surveys on the Pribilof Islands had recorded relatively few Alcids (25% of all carcasses), predominantly murres (55% of Alcidae) and auklets (*Aethia* spp.; 36%). From June 2006 through September 2016, only 6 puffin carcasses had been recorded (**Fig 4**). Within this same baseline period, Procellariiforms were most abundant (56% of the total), mainly Northern fulmars (*Fulmarus glacialis*; 47% of Procellariiformes) and Short-tailed shearwaters (*Ardenna tenuirostris*; 37%; **Fig 4**). Extending baseline comparisons south to the Aleutian Islands: Tufted puffins made up 4–6% of annual encounters, and were almost completely absent during the fall/winter period (**Fig 4**). Crested auklets accounted for 3–16% of carcasses encountered in the Aleutian Islands, and almost all were found in the fall/winter period (**Fig 4**).

Significant necropsy findings included emaciation with severe pectoral muscle atrophy (N = 8, 6 Tufted puffins, 2 Horned puffins). On histopathology, atrophy of fat was the most significant finding. Diagnostic testing via culture and PCR revealed no infectious diseases (e.g., pathogenic bacteria and viruses). Domoic acid was not detected in either of the birds sampled, but trace levels of saxitoxin (3.1 to 9.5 ng/g) were detected in stomach or cloacal contents of all four birds, albeit ~2 orders of magnitude below food safety limits (800ng/g). Elimination rate, and minimum concentration at which marine birds experience negative effects of algal toxins are unknown. Although acute toxicosis or disease was not diagnosed in these birds, these causal factors cannot be entirely ruled out due to the small number of birds tested.

Where molt could be determined from photographic evidence, 95% of the adult Tufted puffins were classified as in flight feather molt (N = 245; **S2 Table**), indicated by wing chord measurements at least 2 cm shorter than the minimum adult wing chord length of 18cm [65]. None of the Horned puffins or Crested auklets were classified as in flight-feather molt. Molt state could only be determined in 3 of the 17 murres (**S2 Table**).

In sum, this MME was characterized by carcass encounter rates 60–80 times higher than baseline, with an unprecedented abundance of adult Tufted puffins, almost all of which were in flight-feather molt and were starving.

### Catchment analyses

**Fig 5:**
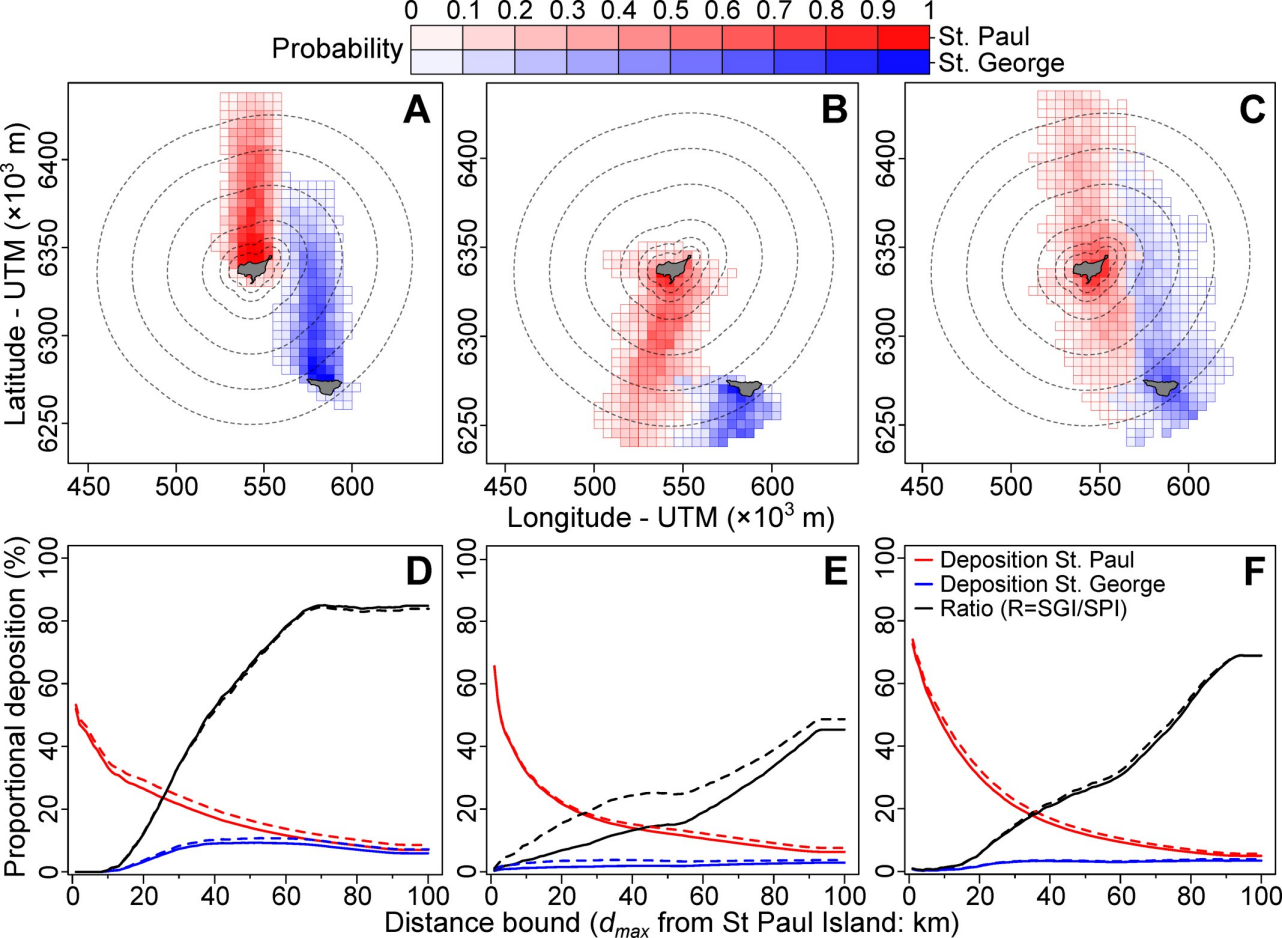
Probability of carcass deposition as a function of location and distance from St. Paul (SPI) and St. George (SGI) Islands, Alaska. Probability of carcass deposition as a function of start location (A-C; median float duration = 7 days), and proportional deposition as a function of the assumed at-sea distribution, proxied by maximum distance, *d*_*max*_, from SPI (D-F), are explored for carcasses deposited between 17 to 21 October 2016 (A & D), 27 October to 1 November 2016 (B & E) and 15 to 23 November 2016 (C & F). Dashed contours in A-C indicate distances of 5, 10, 20, 40, 60 and 80 km from the SPI coastline. Solid/dashed lines in D-F represent results from alternate formulations of float duration (solid = 7 day scenario, dashed = 9 day scenario).

To explore whether carcass deposition could be proxied by wind-driven dispersal of carcasses, we divided deposition modeling into three periods based on occurrence of beached bird surveys (**Table 1**) and prevailing wind direction: period 1 from 17–21 October 2016—daily surveys and predominantly northerly winds; period 2 from 27 October to 1 November 2016—intermittent surveys and predominantly southerly winds; and period 3 from 15–23 November 2016—intermittent surveys and variable northerly winds (**S2 Fig**). After 23 November 2016, surveys were too sporadic due to deteriorating weather, preventing an examination of carcass deposition patterns. During the first period, catchment analysis suggested that the majority (97.5%) of beached birds would likely originate from north of the island (**Fig 5A**) matching the observed pattern of deposition (97% of carcasses observed on North Beach; **Table 1**). By contrast, during the second period, catchment analysis suggested that the majority (95%) of carcasses deposited on SPI likely originated from south of the island (**Fig 5B**), which again mirrored the observed pattern of carcass deposition (**Table 1**). By the third period, the catchment area was less well-defined (**Fig 5C**), with approximately equal proportions of simulated deposition on SPI originating from locations north of the island versus south.

**Fig 6:**
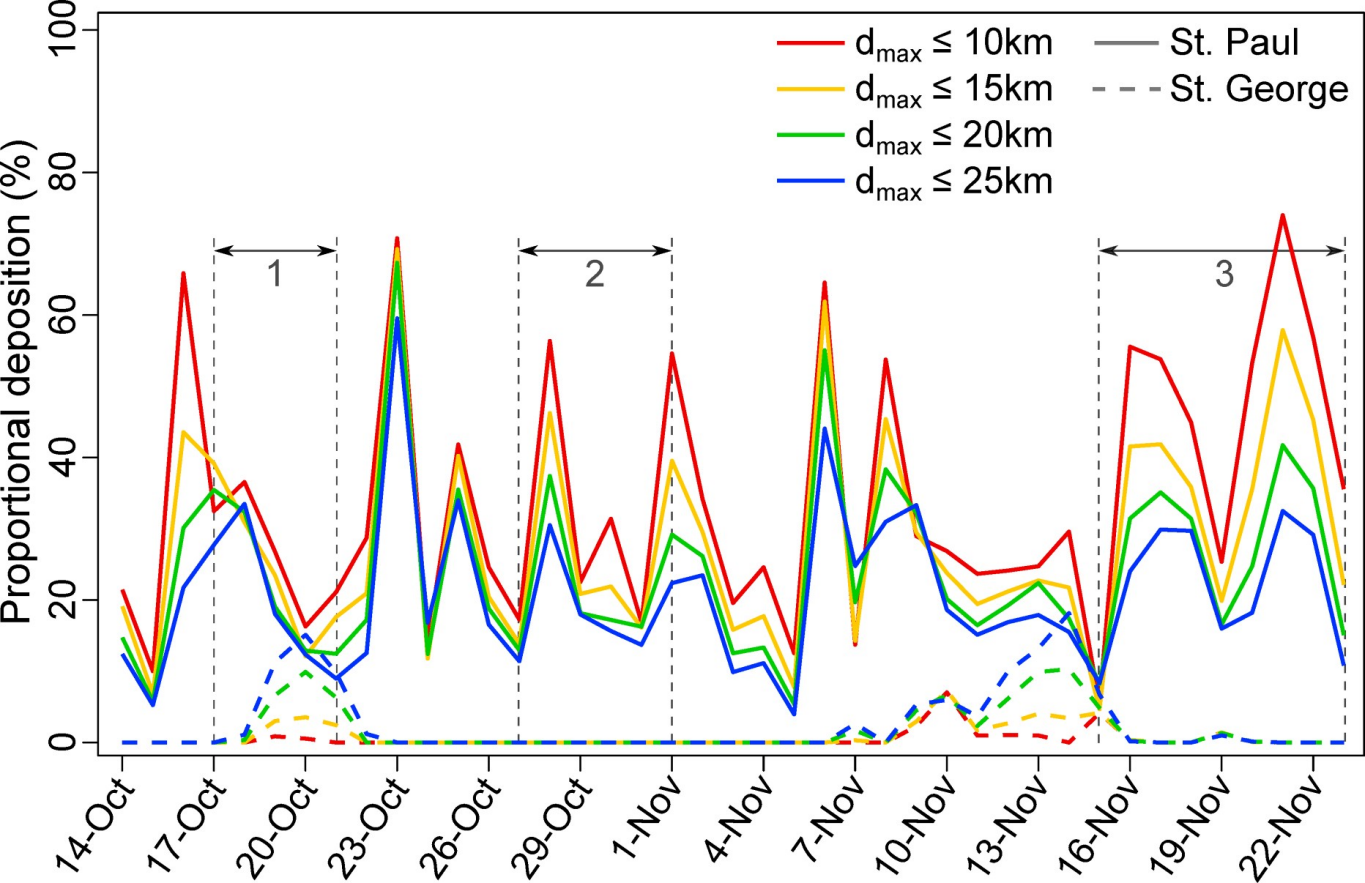
Time-series of simulated deposition (assuming constant mortality per day and median carcass float duration of 7 days) on St. Paul (solid lines) and St. George (dashed lines) Islands, Alaska. Results are shown for four alternate distributions of moribund birds: birds distributed uniformly from St. Paul to 10 km, 15km, 20km and 25 km offshore. Time periods in which deposition was observed on St. Paul are bounded by numbered (1–3 for each deposition window) vertical gray dashed lines.

We used our catchment analysis to explore the maximum distance from SPI moribund or deceased birds might have originated from, given a lack of reported carcasses from SGI. During the first period of deposition on SPI, if moribund birds had been farther than ~19 km from SPI, deposition on SGI should have been ~10% of the SPI rate, rising to 50% for *d*_*max*_ = 38 km (**Fig 5D**). Due to the switch in prevailing wind direction from northerly to southerly during the second period, simulated deposition on SGI was considerably lower, reaching only 10% of the SPI rate at *d*_*max*_ ≈ 30 km (**Fig 5E**). However, there were notable differences between the 7 and 9 day float duration scenario’s, particularly at distances closer to SPI, likely resulting from carcasses floating long enough to reach SGI in the latter scenario (**Fig 5E**). The expected deposition ratio over the third window was intermediate, reaching 10% at *d*_*max*_ = 25 km and 50% at *d*_*max*_ = 77km (**Fig 5F**). Setting the ratio at 20% would result in maximum distances from SPI of 23km, 29km (9d) to 61km (7d) and 37km across time-periods, respectively (**Fig 5D–5F**). Time-series of simulated deposition suggest that if moribund birds were < 20 km from SPI there would have been minimal deposition on SGI overall, albeit with punctuated periods of much higher deposition particularly from the 18 to 21 October and 11 to 15 November (**Fig 6**) when considerable carcass deposition on SPI was observed. If moribund birds had been distributed out to 25 km from SPI, particle simulations suggest there would have been periods of comparable deposition on SGI when deposition was recorded on SPI (**Fig 6**). Combining these findings and the observation that no carcasses were recorded from SGI, we report mortality estimates for distributions with *d*_*max*_ from 2 km (extreme nearshore compression) to 20 km (the most dispersed that maintains minimal deposition on SGI).

### Total mortality estimates

**Fig 7:**
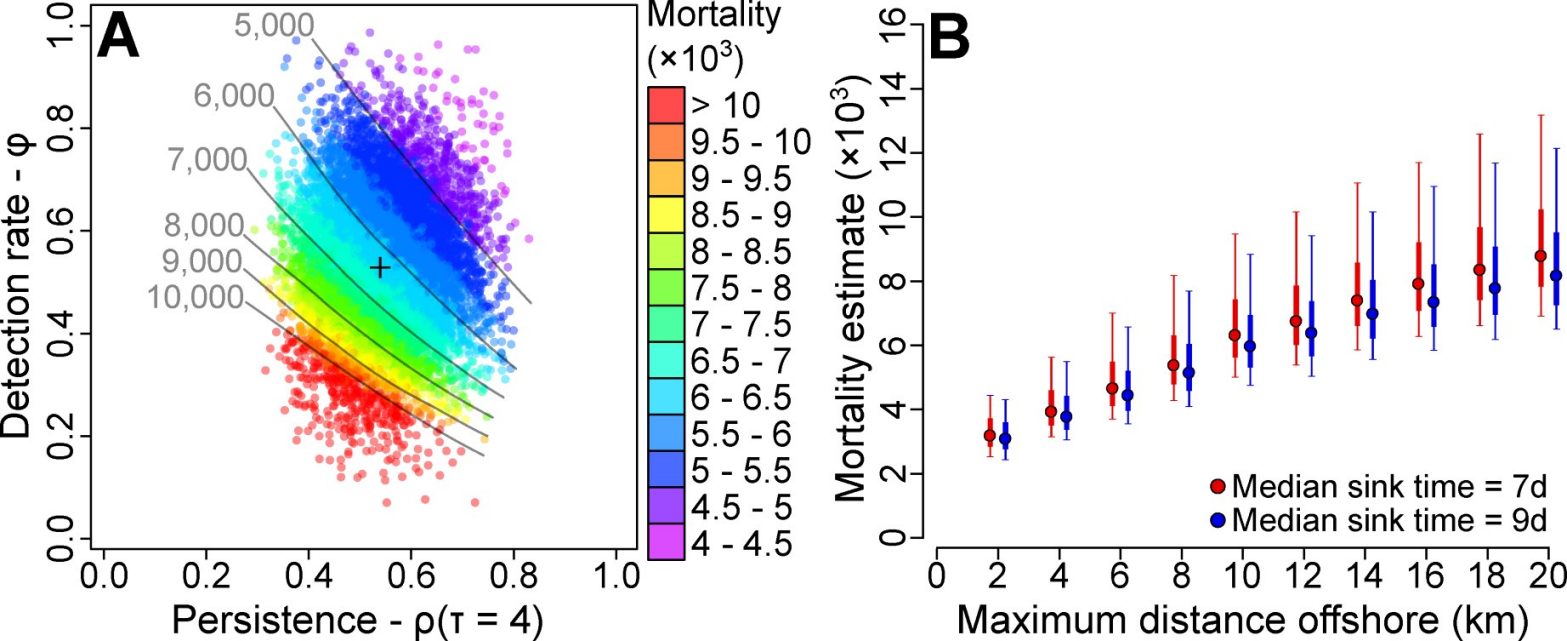
Total mortality estimates for a range of parameter estimates and distribution assumptions. Individual mortality estimates from a single model (A) run assuming moribund birds were distributed uniformly up to 10 km from St. Paul Island, and that carcasses remained afloat for 7 days on average, plotted as a function of detection and persistence rates that were randomly drawn for that model run. Contours represent equal mortality estimates for combinations of detection and persistence rate, and + represents the median value of persistence and detection rate. (B) Median estimates of total mortality for alternate scenarios of spatial distribution (uniform up to a maximum distance offshore of SPI) and float duration (median sink times of 7 and 9 days) plotted as a function of assumed distribution, with 50 (thick lines) and 95% (thin lines) confidence intervals calculated across random draws of persistence and detection rate within each modeled scenario.

Mortality estimates varied considerably as a function of model parameters, ranging from 0.7 to 2.1 (99% range across permutations) times the median value due to alternate values of carcass persistence and detection (**Fig 7A**). For at-sea distributions from nearshore compression (*d*_*max*_ = 2 km from SPI) to the maximum limit suggested by catchment analyses (*d*_*max*_ = 20 km), our median total mortality estimates ranged from 3,150 (*d*_*max*_ = 2 km, 95% CI: 2,415–4,870) to 8,800 (*d*_*max*_ = 20 km, 95% CI: 6,700–15,070) birds (**Fig 7B**). Marginally higher mortality estimates resulted from the assumption that carcasses remained afloat for a median of 7-days compared to 9-days, with the difference increasing from 3.2% for a *d*_*max*_ of 2 km (3,245 versus 3,145, 7-d versus 9-d scenario) to a difference of 7.4% for *d*_*max*_ = 20 km (8,840 versus 8,230) (**Fig 7B**). However, this difference was marginal in comparison to persistence/detection rate uncertainty (upper bound ≈ 2×lower bound), and uncertainty regarding at-sea distribution (upper bound ≈ 2.7×lower bound), likely because simulated carcass deposition occurred relatively soon after release (within 3 days; see **S3 Fig**) within our bounded distributions. As we have no additional information to constrain at-sea distributions, we report estimated mortality of 3,150 to 8,800 birds. Furthermore, given that 87% of the carcasses found from 17 October to 23 November were Tufted puffins, we estimate that 2,740 to 7,600 Tufted puffins died during this time (depending on *d*_*max*_), with outer limits of 2,100 to 13,100 (95% CI).

## Discussion

### Mortality impact

Our analyses suggest that thousands of Alcids, predominantly adult Tufted puffins, died during this event around SPI in the eastern Bering Sea. The Tufted puffin population on the Pribilof Islands has been estimated at only 7,000 breeding individuals (SGI: 6,000, SPI: 1,000) [43], although this estimate is decades old, and may be imprecise due to the relatively inaccessible breeding habitat of this species [66]. However, taken at face value, our most likely mortality estimates represent ~39 to 109% of the Pribilof Island population, making it likely that affected birds also originated from other colonies. Prior to this event, the occurrence of beachcast Tufted puffins was truly rare (0.35% of all carcasses found, N = 283) on the Pribilof Islands. Percentages are slightly higher during the breeding season (June-July) on Aleutian island colonies harboring absolutely larger populations (e.g., 7% of carcass finds on Buldir Island; breeding population ~38,000; **S6 Fig**). Outside of the breeding season, the absence of puffin carcasses from beached bird surveys on the Pribilof Islands, and more generally from beached bird surveys throughout the Bering Sea (**Fig 4**), is likely due to their winter-migration to pelagic waters [67]. Little is known about dispersal patterns of Tufted puffins in the Bering Sea, but it is thought that breeding adults disperse towards wintering grounds throughout the central North Pacific [67] immediately after chicks fledge (late August to early September on Aiktak Island–[68]), such that densities in the SE Bering Sea are minimal by late October [69, 70]. Local and traditional knowledge on SPI also suggests that Tufted puffins leave within a relatively short timeframe from the end of August to mid-September and are rarely seen in October and November. Collectively this information indicates that Tufted puffin distribution was different from usual in the early winter of 2016, and that birds affected by this die-off were not necessarily local breeders. Alternatively, birds dispersing towards the shelf break (**Fig 1**), a productive area [18, 42, 71, 72] favoured by Tufted puffins during the breeding season [73], from Bering Sea colonies farther afield (e.g., east and north of the Pribilof Islands) may have come into close proximity of the Pribilofs during the MME window. Sizeable Tufted puffins colonies are located on Shaiak Island (~80,000 breeders–[74]) in the eastern Bering Sea (**S6 Fig**) and if carcasses washing ashore on SPI included individuals from this colony, our total mortality estimates would correspond to a 3–10% loss in breeding population size. Alternately, if birds observed during the MME included those from the relatively smaller northern breeding populations of Tufted puffins (i.e. St Matthew Island: ~3,500; St Lawrence: ~7,000 –[74]) dispersing southwards, then this event would have been associated with large declines in breeding colony size (26–72% loss). Tufted puffins have sustained dramatic declines in the Gulf of Alaska [75], as well as in British Columbia [76] and on colonies in the northern California Current [77], making this event, although spatially constrained and absolutely small relative to other documented Alcid MMEs [38, 45], of concern. However, because there is no definitive way to assign collected carcasses to their respective colonies, the true population impact of this event remains unknown.

### Mortality estimation

Estimating total mortality for this event was hampered by uncertainty regarding carcass detection and persistence rates, and the at-sea distribution of moribund birds prior to mortality. Employing mark-resighting methods during a mortality event [59], can reduce the uncertainty of mortality estimates by constraining carcass persistence and detection rates, but requires considerable resources and planning, which may not be possible during an ongoing event. However, the largest source of uncertainty in our modeling was in the at-sea distribution of birds, which manifestly altered estimates of the proportion of carcasses that make it to shore [54, 57, 78]. In the absence of other distribution information (i.e. collected from concurrent at-sea surveys [38, 52]), and because carcasses were observed on SPI and not SGI, we made the simplifying, yet limited, assumption that moribund birds were uniformly distributed around SPI out to a distance where deposition on SGI would have been expected. If moribund birds had moved closer to shore in response to mortality inducing conditions, as was potentially the case for the Cassin’s auklet die-off along the California to Washington coast in 2014–15 [38], then observed deposition could have resulted from relatively low mortality (i.e. < 3,000 birds). Why and if apparently starving pelagic marine birds move inshore remains unknown, although this is a precondition for seabird mass mortality events to be captured by beached bird surveys [52, 54, 57]. Further research into the behavioural responses of stressed seabirds, particularly with regards to shoreward migration, would benefit our understanding of seabird mortality events and improve our ability to constrain estimates of their magnitude.

### Event characteristics

The majority of Tufted puffins observed during this event were adults in wing molt, a condition also observed during other puffin mortality events [79]. Tufted puffins, as with other Alcids, undergo nearly synchronous flight feather molt [80], rendering them flightless for up to 40 days [81]. Wing molt is a particularly stressful time for pursuit divers as the growth of new feathers increases nutritional requirements at a time when foraging is constrained [79, 80] and may be more energetically demanding due to reduced wing area [82]. Perhaps because of this, wing molt in Alcids is temporally constricted to post-breeding, post-migration to wintering foraging grounds and prior to the onset of harsh winter conditions [79, 80]. For Tufted puffins, wing-molt is reported to occur between August and October [83], suggesting that affected birds were molting relatively late, although molt phenology is likely later at higher latitudes (e.g. [84]). If birds were in relatively poor body-condition following breeding/post-breeding dispersal and/or if prey was unavailable in the immediate vicinity of SPI, then the additional nutritional requirements due to the loss and subsequent regrowth of flight feathers, coupled with the lack of mobility to find prey elsewhere, likely acted to increase relative mortality of molting birds, contributing to the overall magnitude of this event.

While this die-off was dominated by Tufted puffins, Crested auklet carcasses became increasingly abundant from mid-November onwards. Crested auklets breed throughout the Bering Sea with major colonies in the north (St Matthew, Hall and St Lawrence Islands) and along the Aleutian Island chain [85, 86], in addition to the ~ 34,000 breeding individuals on the Pribilof Islands (SGI: 28,000, SPI: 6,000 –[85]). Unlike Tufted puffins, post-breeding Crested auklets disperse northwards towards foraging grounds close to the ice-edge, before returning south to overwintering locations in the northwest Pacific (Kuril Islands and the sea of Okhotsk) and the southeast Bering Sea, including near the Pribilof Islands [87–89]. Consequently, the later wave of Crested auklet mortality may have been associated with the usual southerly migration of these birds. While Tufted puffin and Crested auklet mortality observed in 2016 was coincident, suggestive of a common causal factor, event-characteristics were species-specific, with molt and altered distribution implicated for Tufted puffins, and elevated post-migration/over-wintering mortality [40] at their usual wintering grounds for Crested auklets.

### Indicators of ecosystem change

Mass mortality events of marine birds are often linked to food stress [38, 90, 91]. Within the Bering Sea, large-scale mortality events in 1983 and 1997 were linked to changes in prey phenology (primarily zooplankton), abundance and composition, as a result of ocean-climate anomalies [1, 34, 36]. Massive shifts in North Pacific marine ecosystems have been observed from 2013 to 2017 as a result of anomalous atmospheric conditions [92], including the sustained presence of the northeast Pacific marine heatwave [10]. Thus far, these shifts have been linked directly to two seabird MMEs [38, 45]. In the Bering Sea, atmospheric conditions from 2014 onwards resulted in decreased winter sea-ice extent and earlier retreat, and associated elevated water temperatures [25, 26]. By 2015/2016, observations were indicative of reductions to forage fish abundance (capelin) and energy density (juvenile Pollock), and reduced abundance of large lipid-rich copepod species and euphausiids on the southern Bering Sea shelf [26, 93]. Duffy-Anderson et al. [26] also reported that the distribution of higher quality prey species (i.e. large lipid-rich copepods and euphausiids) may have shifted northward in the Bering Sea, associated with the retracted cold pool. Of the two species primarily affected by this die-off, Tufted puffins prey on forage fish (i.e. juvenile Pollock, capelin, pacific sandlance) and invertebrates (euphausiids and squid–[67]), whereas Crested auklets are planktivorous, feeding primarily on euphausiids (Thysanoessa spp.–[94]) and large calanoid copepods [95, 96]. As such, Tufted puffins, Crested auklets and other piscivorous or planktivorous seabirds foraging on the southern Bering Sea shelf, may have been subjected to food stress, which in combination with molt for Tufted puffins and southward migration in Crested auklets, may have ultimately caused the documented wave of mortality.

As this die-off didn’t affect the neighbouring island of SGI, it is likely that birds were highly localized to SPI, or that birds were north of SPI, from which expected deposition on SGI would have been minimal (see **Fig 5**). However, this does raise the question of relative susceptibility of seabirds among the Pribilof Islands. Advection of productive oceanic water into the Pribilof domain likely influences productivity and prey availability more strongly at SGI due to its proximity to the shelf break (~ 25 km, compared to 90 km for SPI—[97]), such that foraging conditions near SGI may be more consistent than at SPI [42, 98]. Given overall poorer foraging conditions (i.e. [26, 93, 99]), the relatively stronger influx of oceanic waters at SGI than at SPI may have created differential patterns of food stress, and subsequently mortality. However, given the lack of information regarding the at-sea distribution of birds during the mortality event, we have no way of discerning among patterns in relative abundance versus mortality as the reason for differences in beachings between SPI and SGI.

Although evidence was suggestive of starvation as the primary cause, factors other than prey abundance/quality may have contributed to this mortality event. While weather conditions during the event did not point to storminess as a primary cause (see **S2 Text**), the onset of winter storms would have likely increased energetic requirements (i.e. [100]) and potentially prevented birds from foraging [101], exacerbating conditions, especially if prey quantity/quality was limiting [26]. This may be particularly true towards the end of the mortality event (i.e. Crested auklets) as wind-speeds in January were particularly strong (**S2 Text**). Whether toxigenic algae (e.g., *Pseudo-nitzschia*) was also a contributory factor remains unknown. Although trace levels of saxitoxin were found in all carcasses sampled (n = 4), none were diagnosed with acute toxicosis, suggesting that toxins—if they were a factor—were not primarily responsible. Given warming ocean temperatures and increasing light levels due to northern retreat of sea ice in the Alaskan subarctic/Arctic, increasing prevalence of harmful algal blooms is likely [102]. Thus understanding pathways of ingestion in marine birds, and levels of toxin inducing harm, is critical.

### Conclusions

This mortality event represents one of multiple seabird mortality events that have occurred in the Northeast Pacific from 2014 to 2018 (e.g., [38, 45]), cumulatively suggestive of broad-scale ecosystem change. Although the absolute number of carcass recoveries was small (< 500), total estimated Tufted puffin mortality was in the thousands, and may represent a significant portion of several Bering Sea colonies in addition to Pribilof Islands breeders, which is particularly concerning given recorded declines throughout the southern part of their range [75–77]. Fey et al. [39] suggest that MMEs are indicators of a changing world, and particularly of climate warming. Within the Bering Sea, the occurrence of multi-year stanzas of warm conditions (2001–2005 and 2014–2018; [25, 27]) may be particularly detrimental to seabirds via sustained reductions in the abundance and quality of prey species that were historically abundant [103]. Whether seabirds are resilient to these changes will ultimately govern their long-term viability in an increasingly variable climate.

## Supporting information

S1 Table Survey effort and number of birds found for baseline COASST surveys on St. Paul Island, Alaska relative to the mortality event period in 2016/2017. Data is presented for the calendar months of October to February, and the baseline is presented as the median (med), minimum (min), and maximum (max) of survey effort and counts across years; # = number.
(DOCX)

S2 Table Bird counts summarized by species, age class and primary flight feather molt. (DOCX)

S3 Table COASST survey data summarized on a monthly basis for the Pribilof Islands and the Aleutian Islands, including survey effort, counts and species composition. (XLSX)

S1 Fig Modeled carcass sink functions. Plotted values show the proportion of carcasses remaining afloat as a function of time since death (**A**) and a histogram of the proportion of carcasses that sink as a function of float duration, binned daily (**B**). Black/grey lines/bars, and red lines/bars are for alternate float functions with median durations of 7 and 9 days, respectively.
(TIFF)

S2 Fig Wind speed and direction for St Paul Island during the mortality event. Wind speed and direction are shown for 10 October to 1 November 2016 (**A**) and 2 to 23 November 2016 (**B**). Wind directions are given as daily median (arrow) and 50% range (blue polygons around arrows).
(TIFF)

S3 Fig Histograms of simulated time from release to deposition for particles deposited on St Paul Island for alternate scenarios of at-sea spatial distribution. Histograms show the proportion of particles deposited binned by time afloat (0.5 day bin width) assuming that moribund birds were distributed uniformly up to 10km (**A**), 20 km (**B**) and 80km (**C**) from St. Paul Island.
(TIFF)

S4 Fig Wind rose showing the frequency of wind speed and direction on St. Paul Island, Alaska (57.26°N, 170.19°W) from 1 October to 30 November 2016. Data are 3-hourly averaged wind speed and direction from the North American Regional Reanalysis (NARR) database, and segments are oriented according to incoming wind.
(TIFF)

S5 Fig Carcass persistence as a function of residence time. Plotted are median values as well as 80% and 95% range across 10,000 random draws of persistence function parameters (*ρ*_0_,*ρ*_1_).
(TIFF)

S6 Fig Map of Tufted puffin colonies, color and size-coded according to estimated breeding population size. Colonies referred to in the main text are labelled. SLI: St. Lawrence Island, SMI: St. Matthew Island, SPI: St. Paul Island, SGI: St. George Island, SI: Shaiak Island, EI: Egg Island, KI: Kaligagan Island, AI: Aiktak Island. Data obtained from Alaska Maritime National Wildlife Refuge (AMNWR).
(TIFF)

S1 Text Comparisons of carcass intactness, as a proxy for relative scavenging pressure and satiation, between baseline and die-off beached bird surveys carried out on St Paul Island. (DOCX)

S2 Text Examination of sea surface temperature and indices of storminess prior to and during the 2016 seabird mortality event with respect to local climatology. (DOCX)
