# Supplementary material for: Unusual mortality of Tufted puffins (Fratercula cirrhata) in the eastern Bering Sea
Source: PLoS One. 2019 May 29;14(5):e0216532. doi: 10.1371/journal.pone.0216532 (PMC6541255; doi:10.1371/journal.pone.0216532)
Supplement: S1 Table — Data is presented for the calendar months of October to February, and the baseline is presented as the median (med), minimum (min), and maximum (max) of survey effort and counts across years; # = number. (DOCX) [file pone.0216532.s001.docx]

**S1 Table.** **Survey effort and number of birds found for baseline COASST surveys on St. Paul Island, Alaska relative to the mortality event period in 2016/2017.** Data is presented for the calendar months of October to February, and the baseline is presented as the median (med), minimum (min), and maximum (max) of survey effort and counts across years; # = number.

|  |  | # of surveys conducted | | | Survey effort (km) | | | # birds encountered | | | 2016/17 | | |
| --- | --- | --- | --- | --- | --- | --- | --- | --- | --- | --- | --- | --- | --- |
| Month | # years | med | min | max | med | min | max | med | min | max | # surveys | Effort | # of bird |
| Oct | 10 | 6.5 | 2 | 10 | 6.4 | 1.8 | 10 | 0 | 0 | 2 | 13 | 81.1 | 200 |
| Nov | 10 | 6 | 2 | 10 | 5.9 | 1.8 | 9.8 | 0 | 0 | 1 | 7 | 43 | 125 |
| Dec | 10 | 7.5 | 2 | 13 | 7.3 | 1.8 | 12.6 | 0 | 0 | 5 | 1 | 16.5 | 6 |
| Jan | 9 | 2 | 1.8 | 6 | 2 | 1.8 | 7 | 0 | 0 | 2 | 3 | 19.6 | 26 |
| Feb | 7 | 3 | 1 | 8 | 2.8 | 1 | 8 | 0 | 0 | 1 | 1 | 1 | 2 |
