# Supplementary material for: Unusual mortality of Tufted puffins (Fratercula cirrhata) in the eastern Bering Sea
Source: PLoS One. 2019 May 29;14(5):e0216532. doi: 10.1371/journal.pone.0216532 (PMC6541255; doi:10.1371/journal.pone.0216532)
Supplement: S2 Table — (DOCX) [file pone.0216532.s002.docx]

**S2 Table.** Bird counts summarized by species, age class and primary flight feather molt.

| Species | Juveniles | Adults | | | Age unknown | Total |
| --- | --- | --- | --- | --- | --- | --- |
|  |  | Molting | Not molting | Molt unknown |  |  |
| Tufted Puffin | 15 | 232 | 13 | 9 | 20 | 277 |
| Horned Puffin | 0 | 0 | 15 | 1 | 0 | 16 |
| Common or Thick-billed Murre | 1 | 2 | 1 | 2 | 11 | 17 |
| Crested Auklet | 0 | 0 | 9 | 1 | 31 | 41 |
