# Supplementary figures and images for: Unusual mortality of Tufted puffins (Fratercula cirrhata) in the eastern Bering Sea
Source: PLoS One. 2019 May 29;14(5):e0216532. doi: 10.1371/journal.pone.0216532 (PMC6541255; doi:10.1371/journal.pone.0216532)

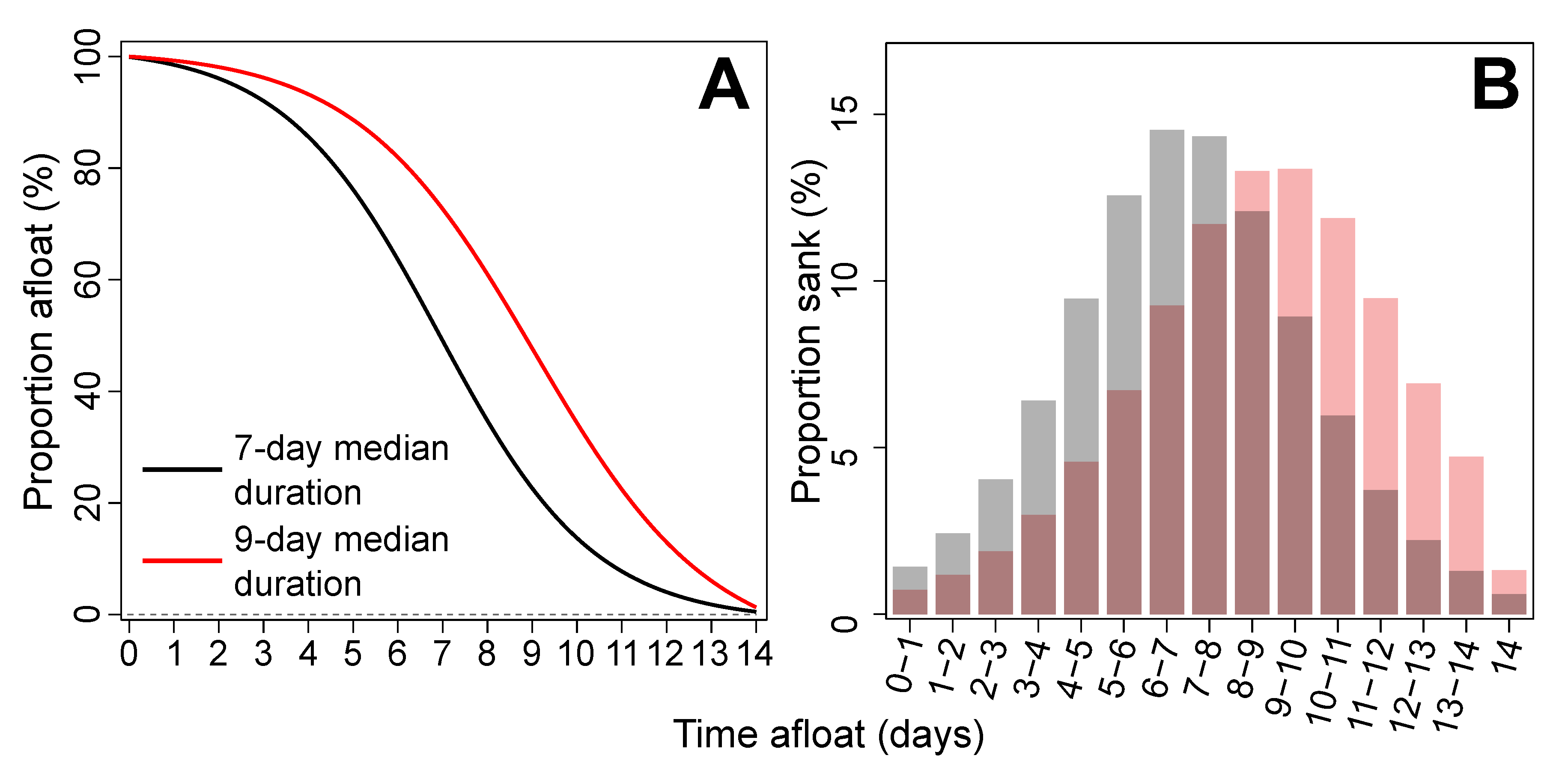

Supplement: S1 Fig — Plotted values show the proportion of carcasses remaining afloat as a function of time since death (A) and a histogram of the proportion of carcasses that sink as a function of float duration, binned daily (B). Black/grey lines/bars, and red lines/bars are for alternate float functions with median durations of 7 and 9 days, respectively. (TIFF) [file pone.0216532.s004.tiff]

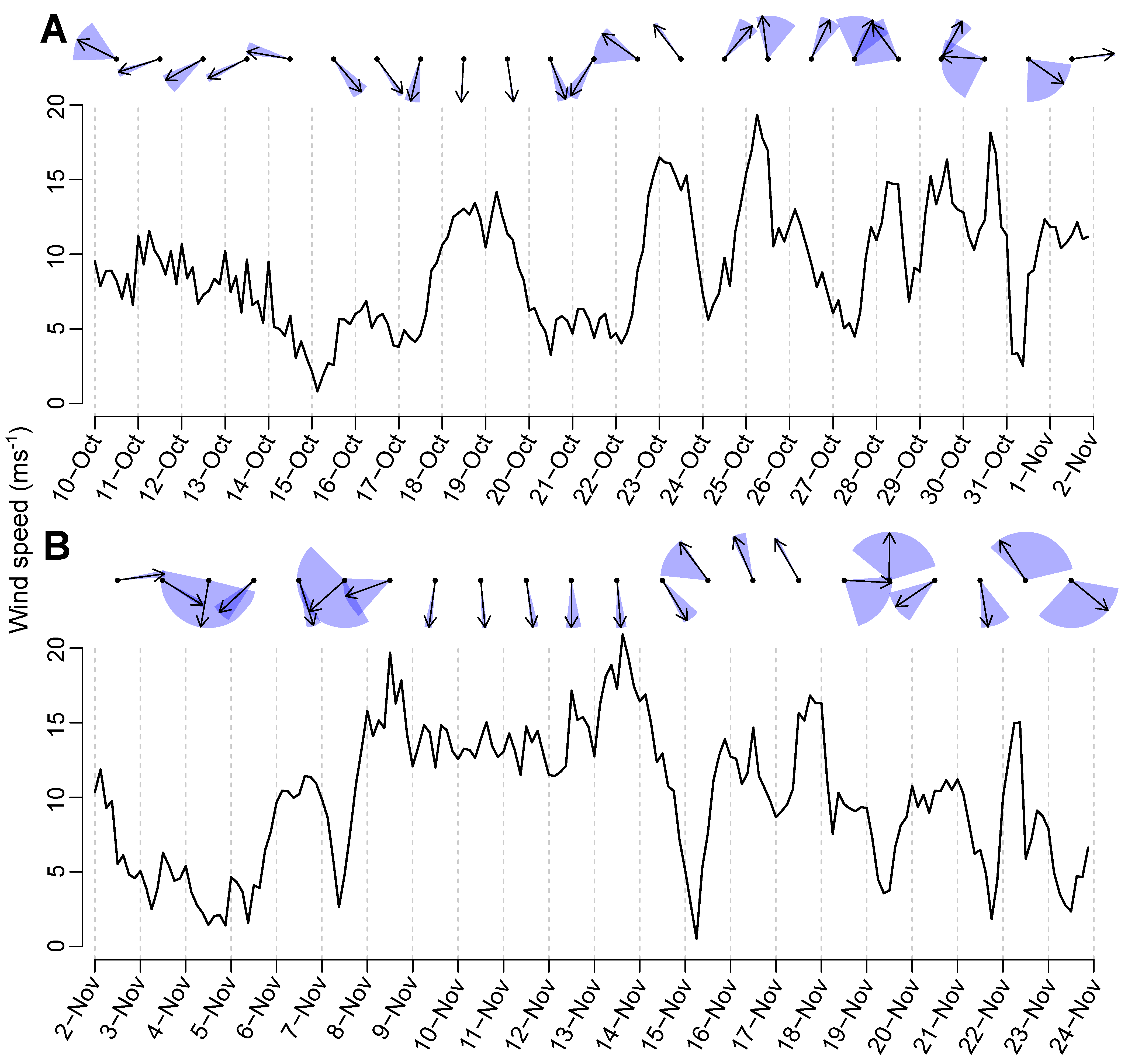

Supplement: S2 Fig — Wind speed and direction are shown for 10 October to 1 November 2016 (A) and 2 to 23 November 2016 (B). Wind directions are given as daily median (arrow) and 50% range (blue polygons around arrows). (TIFF) [file pone.0216532.s005.tiff]

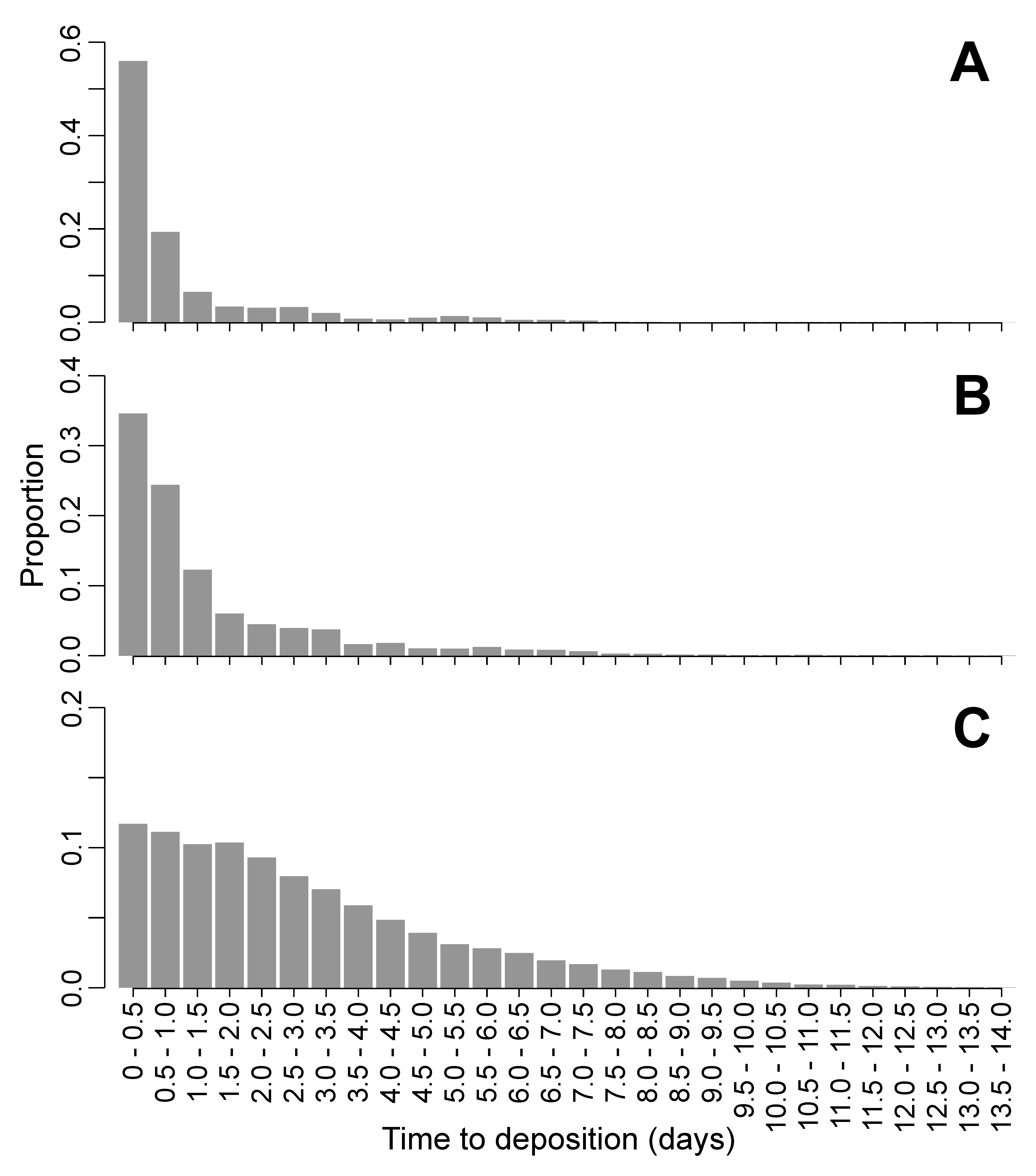

Supplement: S3 Fig — Histograms show the proportion of particles deposited binned by time afloat (0.5 day bin width) assuming that moribund birds were distributed uniformly up to 10km (A), 20 km (B) and 80km (C) from St. Paul Island. (TIFF) [file pone.0216532.s006.tiff]

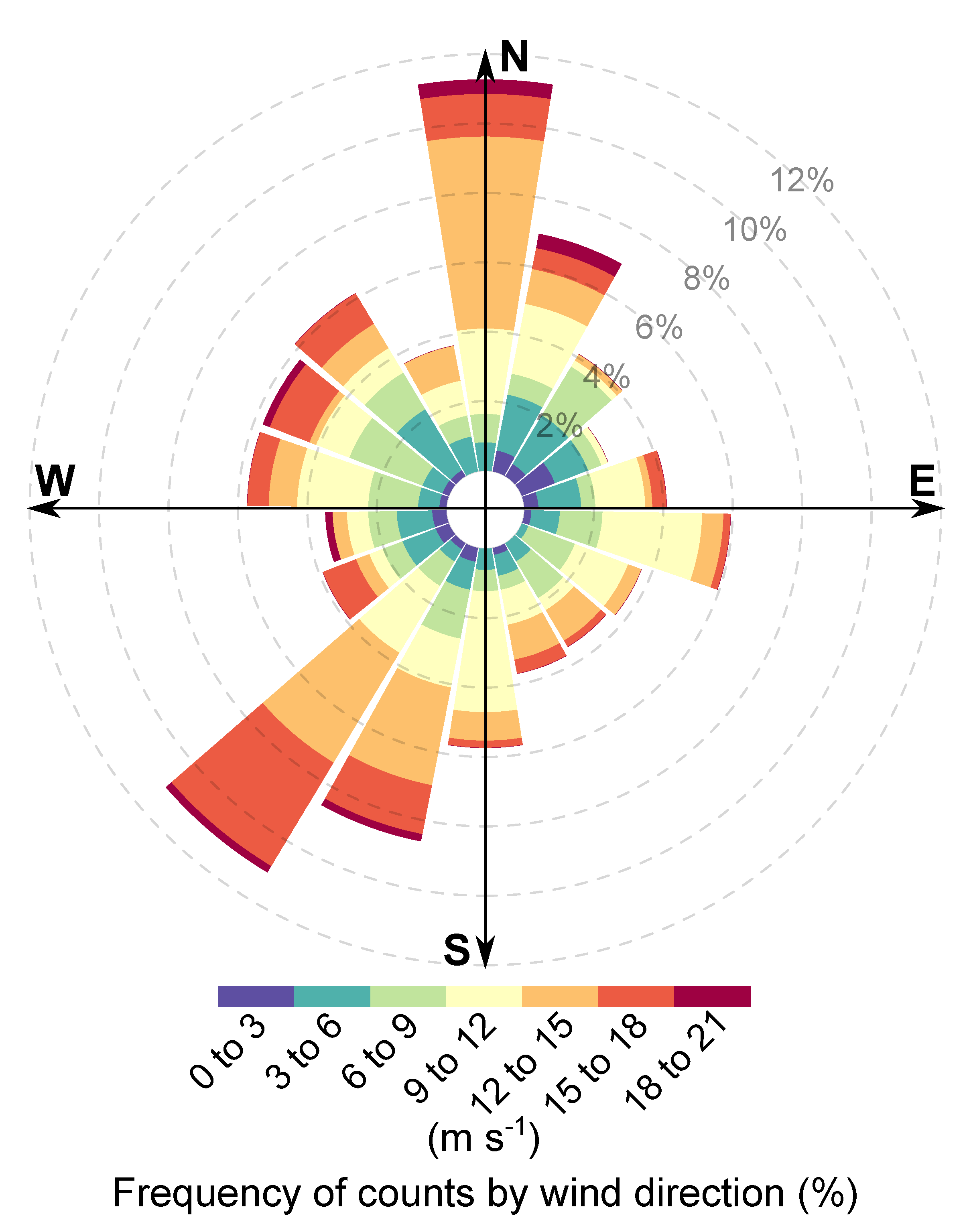

Supplement: S4 Fig — Data are 3-hourly averaged wind speed and direction from the North American Regional Reanalysis (NARR) database, and segments are oriented according to incoming wind. (TIFF) [file pone.0216532.s007.tiff]

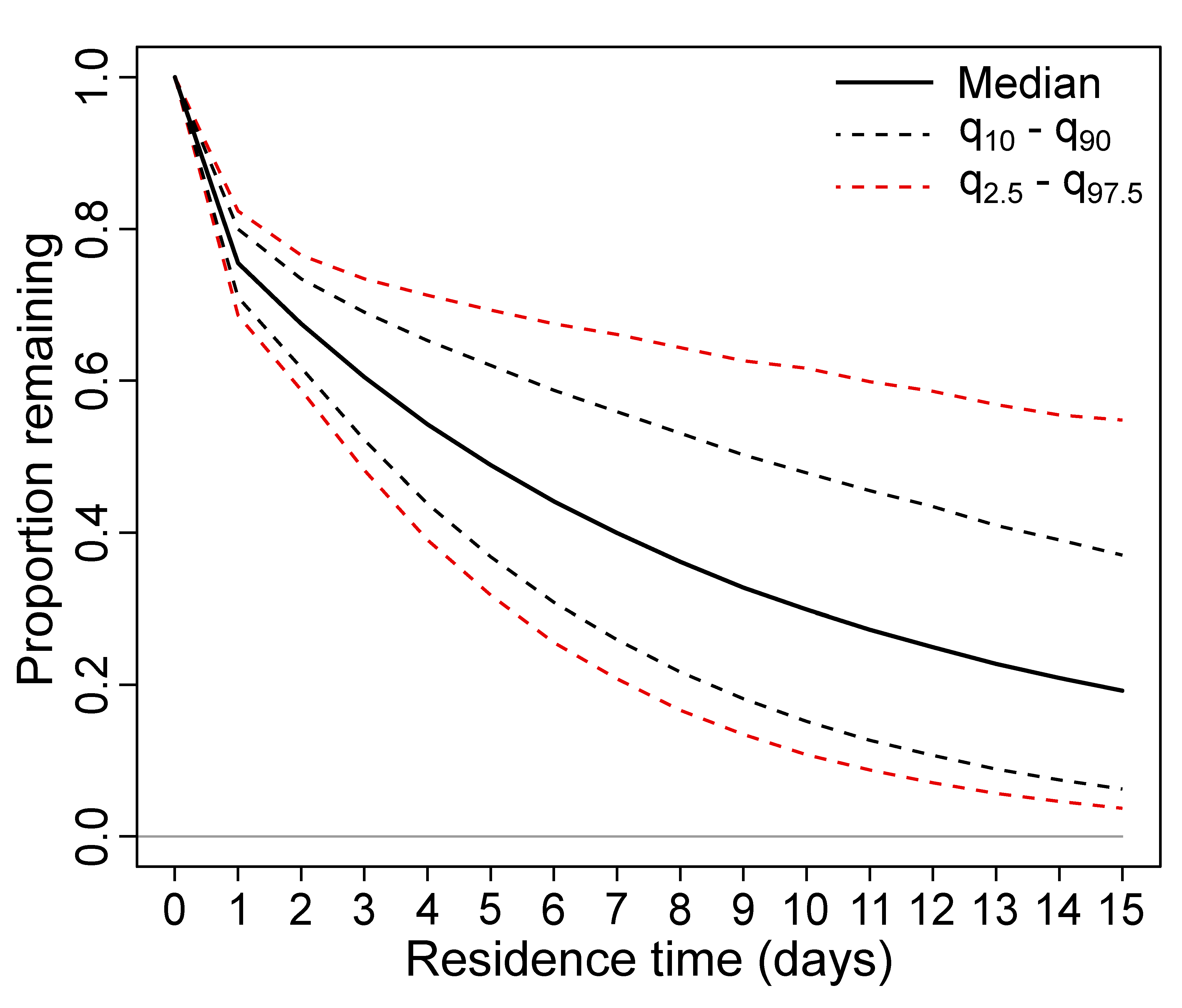

Supplement: S5 Fig — Plotted are median values as well as 80% and 95% range across 10,000 random draws of persistence function parameters (ρ0,ρ1). (TIFF) [file pone.0216532.s008.tiff]

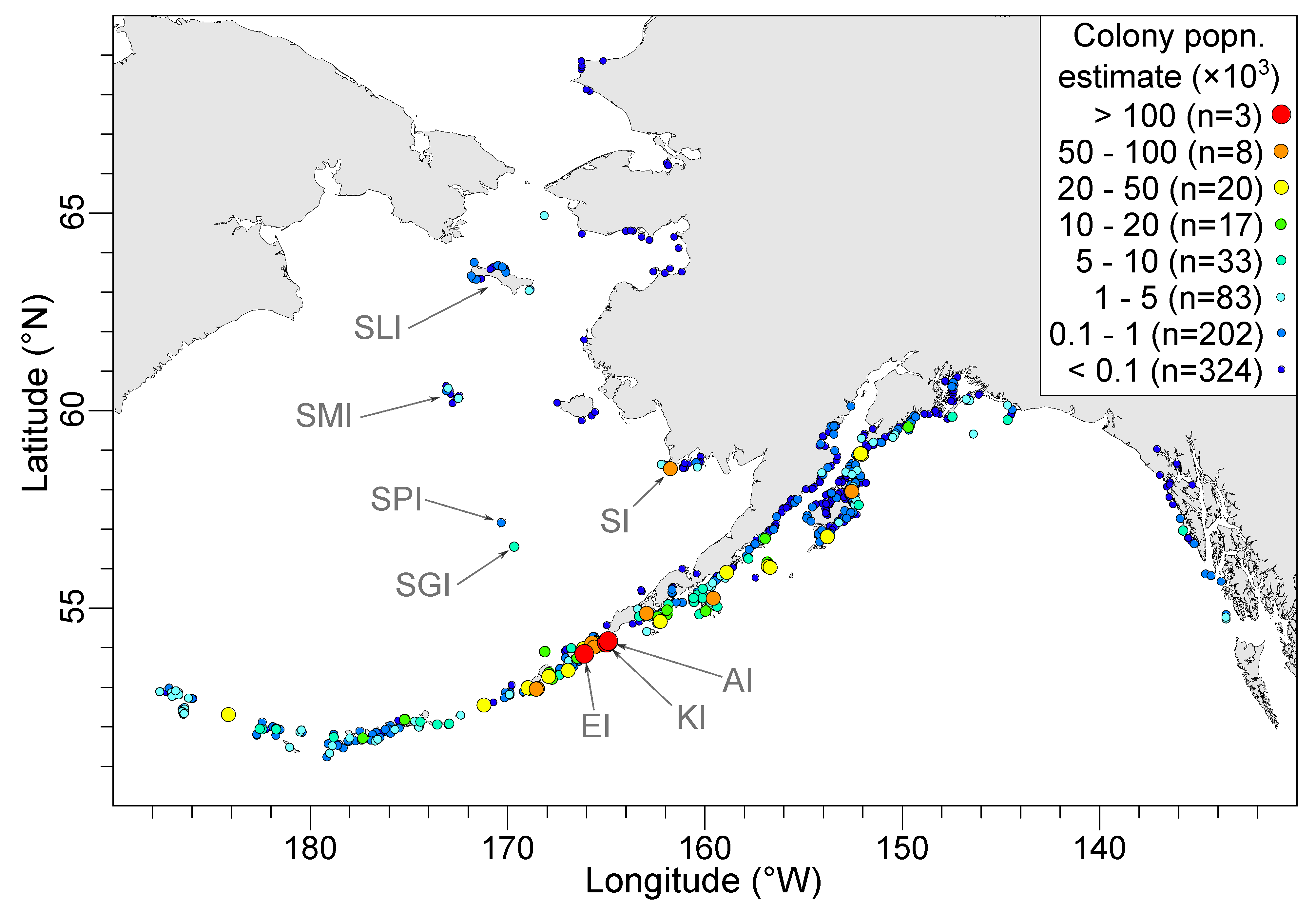

Supplement: S6 Fig — Colonies referred to in the main text are labelled. SLI: St. Lawrence Island, SMI: St. Matthew Island, SPI: St. Paul Island, SGI: St. George Island, SI: Shaiak Island, EI: Egg Island, KI: Kaligagan Island, AI: Aiktak Island. Data obtained from Alaska Maritime National Wildlife Refuge (AMNWR). (TIFF) [file pone.0216532.s009.tiff]
