# Supplementary material for: Unusual mortality of Tufted puffins (Fratercula cirrhata) in the eastern Bering Sea
Source: PLoS One. 2019 May 29;14(5):e0216532. doi: 10.1371/journal.pone.0216532 (PMC6541255; doi:10.1371/journal.pone.0216532)
Supplement: S1 Text — (DOCX) [file pone.0216532.s010.docx]

**S1 – Supplementary text**

*Jones et al. (2019) Unusual Mortality of Tufted puffins (Fratercula cirrhata) in the eastern Bering Sea. PLoS ONE.*

***Intactness Analyses***

To explore whether island scavengers, primarily Arctic foxes (*Vulpes lagopus*), were satiated by the magnitude of carcass deposition/availability during the event, we tested and compared models of carcass intactness coded as a binomial response; 1 = fully intact; 0 = any wounds/partial carcasses; where the latter was assumed to be an indication of scavenging. In addition to a temporal factor distinguishing birds found during baseline surveys (prior to October 2016) from MME birds (October 2016 to February 2017), we trialed predictors of: carcass abundance specific to survey and bird size, where the latter was divided into three categories by species after Ford 2006 - small: storm-petrels (*Oceanodroma* spp.), auklets (*Aethia* spp.), kittiwakes (*Rissa* spp.) and shorebirds; medium: shearwaters (*Ardenna* spp.), Northern fulmars (*Fulmarus glacialis*), murres (*Uria* spp.) and puffins (*Fratercula* spp.); large: gulls (*Larus* spp.) and cormorants (*Phalacrocorax* spp.). All models included a random-effect of survey to account for similarity of carcass condition that might arise from similar deposition/residence time (i.e. synchronous deposition of multiple carcasses), and scavenging pressure experienced by carcasses found within the same survey. We explored all predictor combinations using binomial generalized linear mixed-effects models (GLMMs) and subsequently ranked models based on small sample size corrected Akaike Information Criterion (AICc).

During the die-off, a large proportion of the carcasses encountered were fresh (e.g., intact carcass with clear fully rounded eyes). Our best-fitting model describing carcass intactness included time period (baseline versus event) as the single predictor (**Table 1**), highlighting that many more carcasses were found intact during this event (74.5%, 95% CI: 60-85%) than in baseline surveys (17%, 95% CI: 10-25%). The second ranked model (Δ_AICc_ of < 2.0) also included a significant positive effect of carcass abundance, indicating that intactness was higher in surveys with more carcasses.

These results suggest that the island's scavenger population may have been satiated by carcass deposition during the event, and possibly more so during periods with highest carcass abundance. However, because we cannot account for differences in survey frequency between baseline (search interval: 2-4 weeks) and event (daily) surveys in these models, these differences may to some unknown degree be due to the increased survey frequency during the event, and therefore we cannot conclusively report that the difference in intactness was due to scavenger satiation.

**Table 1. Model results of binomial GLMMs (link = logit) fitted to carcass intactness data.**

| Rank | Model stats | | Parameter estimates | | | |
| --- | --- | --- | --- | --- | --- | --- |
|  | AICc | Δ_AICc_ | Int. | Abun. | Size | Time |
| **1** | **632.0** | **0** | **1.07^a^** |  |  | **-2.63** |
| **2** | **633.2** | **1.3** | **0.67^a^** | **0.02** |  | **-2.29** |
| 3 | 635.9 | 3.9 | 1.17^b^ |  | M:-0.08, S:-0.20 | -2.63 |
| 4 | 637.2 | 5.3 | 0.80^b^ | 0.02 | M:-0.12, S:-0.18 | -2.30 |
| 5 | 646.7 | 14.8 | -1.75 | 0.11 |  |  |
| 6 | 650.8 | 18.8 | -1.77^c^ | 0.11 | M:0.03, S:0.00 |  |
| 7 | 662.7 | 30.7 | -1.36 |  |  |  |
| 8 | 666.2 | 34.3 | -1.69^c^ |  | M:0.44, S:0.17 |  |
| Rank | Fitted intactness rate (%) [95% CI] | | | | | |
| 1 | Base: 17% [10-25%], Event: 74% [60-85%] | | | | | |
| 2 | Base (Abun. = 2): 17% [11-24%], Base (Abun. = 10): 20% [12-30%]  Event (Abun. = 2): 66% [43-84%], Event (Abun. = 10): 71% [55-84%] | | | | | |

Values represent model regression or factor coefficients (link scale) of predictors included in that model. Models are listed from lowest AICc to highest, and rows in bold indicate models with ΔAICc < 2. Fitted effects and their bootstrapped 95% confidence intervals are presented for models with ΔAICc < 2.

^a^ Time coefficients represent the difference between baseline birds and event birds, with the coefficient for event birds given as the intercept.

^b^ For models with time and size effects the intercept represents the estimated value for Size=Large and Time=Event birds, with size and time modifiers given in the corresponding columns.

^c^ For models with size effects the intercept represents the coefficient for large birds, and the relative difference between large birds and medium/small birds given in the Size column.

Ford RG. Using beached bird monitoring data for seabird damage assessment: the importance of search interval. Mar Ornithol. 2006; 34: 91-8.
