# Supplementary material for: Unusual mortality of Tufted puffins (Fratercula cirrhata) in the eastern Bering Sea
Source: PLoS One. 2019 May 29;14(5):e0216532. doi: 10.1371/journal.pone.0216532 (PMC6541255; doi:10.1371/journal.pone.0216532)
Supplement: S2 Text — (DOCX) [file pone.0216532.s011.docx]

**S2 – Supplementary text**

*Jones et al. (2019) Unusual Mortality of Tufted puffins (Fratercula cirrhata) in the eastern Bering Sea. PLoS ONE.*

***Environmental framing***

To examine environmental forcing factors during the event we examined information on sea surface temperature and wind speed to examine whether the die-off in 2016 was preceded by extremes of temperature that may alter productivity and availability of seabird prey, and also to identify whether mortality could have been caused to extreme or extended storm events. We used daily sea surface temperature anomaly (SSTa – relative to the 1971-2000 climatology) maps from the NOAA Optimum Interpolation SST V2 High-Resolution data repository (Reynolds et al. 2007) to calculate average SSTa within the eastern Bering Sea, defined as the area from the Aleutian Islands chain north to the Bering Strait and east of 180°W (see main text: Fig 1A), from 2005 through 2016. To examine the influence of weather we created indices of storminess from daily maps of wind speed, w, from the North American Regional Reanalysis (NARR) dataset for the Pribilof Islands (grid cells with latitude: 56 – 58° N, longitude: 172 – 168° W) as follows. For the months of September to December from 2005 to 2016 we calculated average daily wind speed ($\bar{w}$), and the number of days where w ≥ 15 ms^-1^. This upper threshold was defined based on the wind-speed spectrum for September to January, with wind speeds ≥ 15ms^-1^ occurring only 5% of the time during these months from 2005 to 2016 and therefore indicative of extreme conditions. We also include a similar index, but for a lower wind-speed threshold of 10 ms^-1^, as critical wind-speeds above which seabirds are affected by storm conditions are not known, and this allows us to explore a broader range of environmental forcing.

Records of SSTa indicate the eastern Bering Sea was warmer than normal from 2014 to early 2017, with particularly warm episodes from July to October 2014 and May to September 2016 (SSTa in excess of +2°C relative to the 1971-2000 climatology; **Fig 1**). The latter of these two warming episodes is of particular interest as it occurred directly prior to the start of the MME recorded on SPI. Wind speeds for September to December 2016 were within normal limits compared to a baseline inclusive of 2005-2015, with the exception of December 2016, which recorded the highest number of days with wind-speeds ≥ 15 ms^-1^ (4 as opposed to a mean of 2.1; **Table 1**). However, December high wind speeds occurred largely after the estimated timeframe of the die-off, but may have contributed to the latter part of the mortality event that was characterized by deposition of Crested auklets.

**Table 1. Summary indices of wind speed from the North American Regional Reanalysis (NARR) dataset for the Pribilof Islands.** For baseline years the mean, median, min and max are calculated across years for each calendar month.

| Month | Baseline (2005-2015) | | | | 2016 | Rank (2016) |
| --- | --- | --- | --- | --- | --- | --- |
|  | Mean | Median | Min | Max |  |  |
| *Average wind speed -* $\bar{w}$ | | | | | | |
| 9 | 8.2 | 8.1 | 6.3 | 10.1 | 8.3 | 5 |
| 10 | 8.2 | 8.2 | 6.1 | 10.2 | 9.0 | 4 |
| 11 | 9.7 | 9.7 | 8.4 | 11.8 | 8.6 | 10 |
| 12 | 9.2 | 9.0 | 7.7 | 11.1 | 9.8 | 4 |
| *Number of days w > 10ms-1* | | | | | | |
| 9 | 8.8 | 10 | 1 | 16 | 9 | 6 |
| 10 | 9.6 | 9 | 2 | 16 | 12 | 4 |
| 11 | 14 | 13 | 9 | 21 | 13 | 6 |
| 12 | 12.3 | 11 | 7 | 18 | 13 | 6 |
| *Number of days w > 15ms-1* | | | | | | |
| 9 | 1.1 | 1 | 0 | 4 | 2 | 3 |
| 10 | 0.8 | 1 | 0 | 3 | 1 | 5 |
| 11 | 2.4 | 3 | 0 | 4 | 2 | 8 |
| 12 | 2.1 | 2 | 0 | 3 | 4 | 1 |


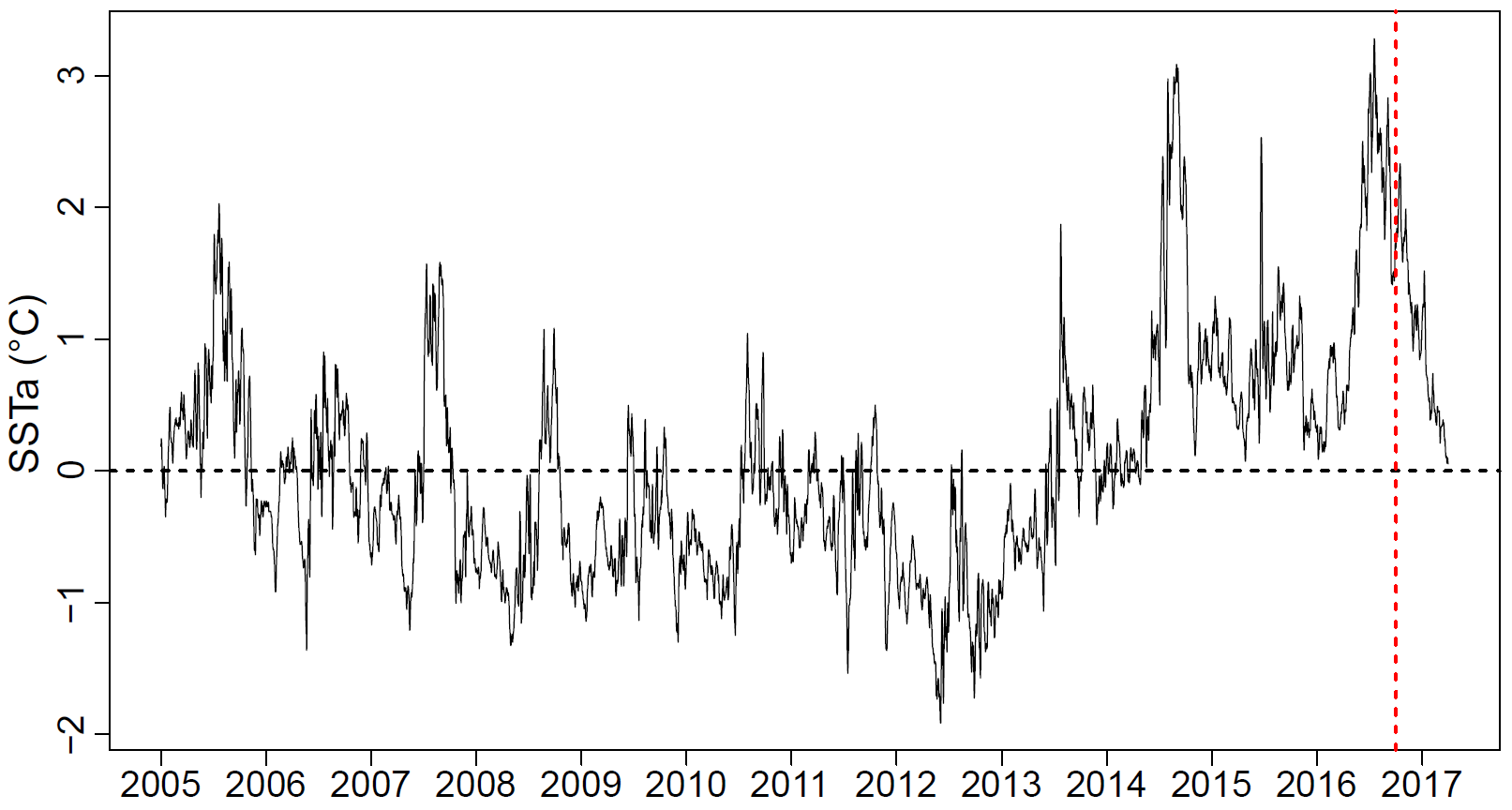


**Fig 1. Time-series of average sea surface temperature anomaly (SSTa) for the eastern Bering Sea.** SSTa data was obtained from the Optimum Interpolation SST V2 High Resolution data repository. The dashed horizontal black line indicates 0°C; dashed vertical red line indicates October 2016.

Reynolds RW, Smith TM, Liu C, Chelton DB, Casey KS, Schlax MG. Daily high-resolution-blended analyses for sea surface temperature. J Clim. 2007; 20(22): 5473-5496.
